# Regulation of Carbon Metabolism by Environmental Conditions: A Perspective From Diatoms and Other Chromalveolates

**DOI:** 10.3389/fpls.2020.01033

**Published:** 2020-07-16

**Authors:** Hélène Launay, Wenmin Huang, Stephen C. Maberly, Brigitte Gontero

**Affiliations:** ^1^BIP, Aix Marseille Univ CNRS, BIP UMR 7281, Marseille, France; ^2^Key Laboratory of Aquatic Botany and Watershed Ecology, Wuhan Botanical Garden, Center of Plant Ecology, Core Botanical Gardens, Chinese Academy of Sciences, Wuhan, China; ^3^UK Centre for Ecology & Hydrology, Lake Ecosystems Group, Lancaster Environment Centre, Lancaster, United Kingdom

**Keywords:** Calvin cycle, CO_2_ concentrating mechanism, *Phaeodactylum tricornutum*, redox regulation, *Thalassiosira pseudonana*

## Abstract

Diatoms belong to a major, diverse and species-rich eukaryotic clade, the Heterokonta, within the polyphyletic chromalveolates. They evolved as a result of secondary endosymbiosis with one or more Plantae ancestors, but their precise evolutionary history is enigmatic. Nevertheless, this has conferred them with unique structural and biochemical properties that have allowed them to flourish in a wide range of different environments and cope with highly variable conditions. We review the effect of pH, light and dark, and CO_2_ concentration on the regulation of carbon uptake and assimilation. We discuss the regulation of the Calvin-Benson-Bassham cycle, glycolysis, lipid synthesis, and carbohydrate synthesis at the level of gene transcripts (transcriptomics), proteins (proteomics) and enzyme activity. In contrast to Viridiplantae where redox regulation of metabolic enzymes is important, it appears to be less common in diatoms, based on the current evidence, but regulation at the transcriptional level seems to be widespread. The role of post-translational modifications such as phosphorylation, glutathionylation, etc., and of protein-protein interactions, has been overlooked and should be investigated further. Diatoms and other chromalveolates are understudied compared to the Viridiplantae, especially given their ecological importance, but we believe that the ever-growing number of sequenced genomes combined with proteomics, metabolomics, enzyme measurements, and the application of novel techniques will provide a better understanding of how this important group of algae maintain their productivity under changing conditions.

## Introduction

**Figure 1:**
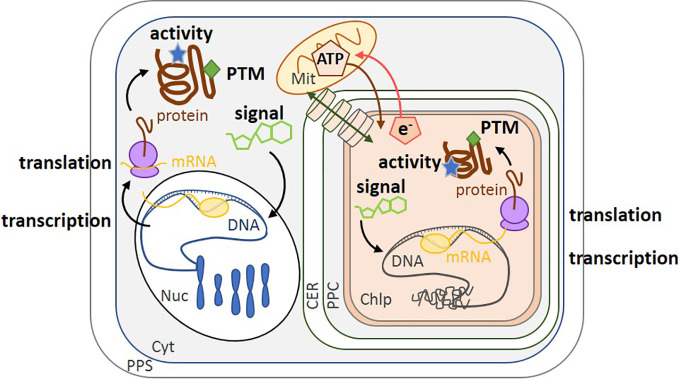
Schematic of the different levels of regulation in diatoms. Transcription of the genome by mRNA polymerase (yellow circle) converts nuclear DNA (blue) and chloroplast DNA (gray) into mRNA (yellow). Translation by the ribosome (purple) converts mRNA into protein (brown). Regulation can also be modulated by enzyme activity (blue stars), post-translational modification (green diamonds) and small molecules such as co-factors or metabolites (green carbon skeletons). The transport of molecules between the cytoplasm and the chloroplast is represented by a green double-headed arrow across cylinders. ATP synthesized in the mitochondrion can be transported into the chloroplast (brown arrow), while the reducing power (e.g., NADPH, represented by e^-^) of the chloroplast is transported to the mitochondrion (red arrow) (Bailleul et al., 2015). PPS, Periplasmic Space; CER, Chloroplast Endoplasmic Reticulum; PPC, Periplastidial Compartment; Mit, Mitochondrion; Chlp, Chloroplast; Cyt, Cytoplasm; PTM, Post-Translational Modification; Nuc, Nucleus.

The chromalveolates are a polyphyletic eukaryote supergroup that includes many photosynthetic lineages including the cryptomonads, dinoflagellates, haptophytes, and heterokonts (also called stramenopiles) (Keeling, 2009). The phylogeny of diatoms and their allied groups is complicated (Dorrell et al., 2017; Falciatore et al., 2020) and while the chromalveolates are not now regarded as a natural group we have retained the name here as a convenient and widely-used term. It has been estimated that over 50% of all formally described protists are chromalveolates (Cavalier-Smith, 2004; Cavalier-Smith and Chao, 2006). Within the diverse clade Heterokonta, diatoms (Bacillariophyceae) are photosynthetic microalgae that comprise between 30,000 and 100,000 species (Mann and Vanormelingen, 2013). They evolved about 250 Myrs ago (Medlin, 2016), are found today in all aquatic environments, and contribute about 20% to global primary production (Falkowski et al., 1998). Like other heterokonts, diatoms originated *via* serial endosymbioses (Stiller et al., 2014) and their chloroplasts derive from a red and a green algal endosymbiosis and also contain genes from prokaryotes, their eukaryotic host, and genes acquired by horizontal transfer (Moustafa et al., 2009; Deschamps and Moreira, 2012; Dorrell et al., 2017). Consequently, diatom genomes are enriched in genes from different origins and this combination has gifted them with unique metabolic features. In addition to the metabolism needed to produce a silica cell wall (Hildebrand et al., 2018) a functioning urea cycle is present (Allen et al., 2011; Nonoyama et al., 2019). Diatoms have an Entner-Doudoroff glycolytic pathway (Fabris et al., 2012) but lack the oxidative pentose phosphate (OPP) pathway in their chloroplast (Wilhelm et al., 2006; Kroth et al., 2008; Gruber et al., 2009). Their principal storage compound is a polysaccharide, chrysolaminarin (β-1,3 linked glucan) that is located in the vacuole rather than the chloroplast (Huang et al., 2018). Diatoms also have a large diversity of the metalloenzyme carbonic anhydrase (CA) that interconverts CO_2_ and HCO_3_^−^. They possess seven of the eight known CA sub-classes, some of which can make use of metal cations other than the canonical zinc (Jensen et al., 2019a; Alissa et al., 2020; Morel et al., 2020). In diatoms, both the large and the small subunits of ribulose bisphosphate carboxylase-oxygenase (RuBisCO) are encoded by the chloroplast genome, in contrast to Viridiplantae where the small subunit is a nuclear encoded protein (Oudot-Le Secq et al., 2007). Moreover, most diatom plastid genomes, unlike those in Viridiplantae and the diatom *Seminavis robusta*, lack introns (Brembu et al., 2014). Also in diatoms, RuBisCO activation is mediated by the protein CbbX (Mueller-Cajar et al., 2011) that does not possess the cysteine residues found in RuBisCO activase (RCA) found in Viridiplantae, and thus cannot be redox regulated (Jensen et al., 2017). In addition, diatoms also have a pigment composition that substantially differs from plants (Carreto and Catoggio, 1976; Falkowski and Owens, 1980; Gilstad et al., 1993; Kuczynska et al., 2015). The most important accessory pigments in diatoms are fucoxanthin and chlorophyll *c* rather than chlorophyll *b* in Viridiplantae (Green, 2011). Also, like all photosynthetic eukaryotes and cyanobacteria, they contain xanthophylls that are derived from β carotene but in contrast, lack the α-carotene pathway. Diatoms are able to acclimate to a broad range of light irradiance and nutrient concentrations by adjusting their physiology and biochemical activity (Schoefs et al., 2017; Heydarizadeh et al., 2019). This requires a variety of mechanisms for balancing energy harvesting and light-energy consuming metabolic processes including carbon fixation (Wilhelm et al., 2006). In contrast to Viridiplantae, diatoms have a very low cyclic electron flow. To equilibrate the ratio of ATP to NADPH required for optimal photosynthesis, the chloroplast and the mitochondrion, that are physically in contact, exchange these compounds (Bailleul et al., 2015) (**Figure 1**).

Regulation can act on, and be studied at, a cascade of different levels from DNA (genomics), mRNA (transcriptomics), proteins (proteomics and post-translational modifications, PTMs), metabolites (metabolomics), and enzyme activity, because each approach provides different types of information (**Figure 1**). Genomes are powerful resources to determine if a specific gene is present while transcriptomics detect under what conditions it is expressed. Fully annotated diatom genome sequences are only available for *Thalassiosira pseudonana*, a marine centric diatom and *Phaeodactylum tricornutum*, a marine or coastal pennate diatom (Armbrust et al., 2004; Bowler et al., 2008). Other sequences are also available for *Fragilariopsis cylindrus* (Mock et al., 2017) and *Pseudo-nitzschia multiseries* (https://genome.jgi.doe.gov/portal/pages/tree-of-life.jsf) and there are further published genomes that are not yet publicly available (Tanaka et al., 2015; Traller et al., 2016; Basu et al., 2017; Villain et al., 2017; Ogura et al., 2018). Lauritano et al. (2019) reviewed the current development of omics approaches on microalgae. Of the 26 eukaryotic microalgal genomes they listed, 15 were from chromalveolates and of these, 8 were from diatoms. However, regulation should also be analyzed at the level of enzyme activity and/or metabolites (metabolomics), as these represent the final outcome of gene expression and activity (Prosser et al., 2014). The lifetime of an active enzyme, or of a metabolite, is related to its rate of synthesis and turnover. While the synthesis rates are on the order of 4–6 amino acids per second for enzymes (Stein and Frydman, 2019) and several seconds for metabolites (Nikolaev and Lohse, 2006), their turnover rates can vary from a few seconds to extended periods respectively. Enzyme activity is also modulated by PTMs, such as acetylation, phosphorylation, methylation, glycosylation and formation/dissociation of disulfide bonds. These types of modulation are very fast (rate on the order of few per second), reversible, and are the most flexible regulatory responses at the protein level (Prabakaran et al., 2012). In diatom RuBisCO, a number of post-translational modifications of the large subunit are present, including 4-hydroxyproline, β-hydroxyleucine, hydroxylated and nitrosylated cysteine, mono- and dihydroxylated lysine, and trimethylated lysine (Valegard et al., 2018). Nevertheless, in order to understand the full scope of regulation by post-translational modifications (Grabsztunowicz et al., 2017) in chromalveolates, more studies are needed on PTM and proteomics. Regulation of gene expression is itself dependent on earlier response regulators (for example, PTMs of histones and transcription factors) and on metabolite productions (for example, cAMP). As a consequence, upon environmental changes, regulation of gene expression occurs over a longer timescale of several minutes to hours (Chauton et al., 2013).

Relationships between mRNA level and protein expression can be observed though this might be influenced by biological (e.g., properties of mRNA and proteins, cell cycle status) and by technical problems (accurate quantification of these two biological molecules) (Maier et al., 2009; Ponnala et al., 2014). Therefore, there are discrepancies in the literature as regard to the extent of correlation between them. Net mRNA levels can be a major contributor to protein abundance, and for instance, positive relationship has been observed in yeast (Fournier et al., 2010), in the green alga *Chlamydomonas reinhardtii* (Castruita et al., 2011) and specifically in the diatom *T. pseudonana* (Clement et al., 2017b). Nevertheless, there are additional mechanisms, that control protein abundance including translational control and differential protein and mRNA degradation rates (Ponnala et al., 2014). However, since data on protein expression and activity are scarce, we have supplemented this type of information with data on gene regulation as a first step to assess how diatoms respond to environmental change, even though there is not always a direct and positive relationship between mRNA level, protein expression and finally, metabolic activity (**Figure 1**).

## Regulation of Photosynthesis by Light and Dark

Changing light levels affect many processes, including cell division, and diatoms can acclimate efficiently to light variation by altering the expression of different cell cycle genes such as cyclins and cyclin-dependent kinases genes (Huysman et al., 2013). Here, we focus on the best-studied effect of light, the regulation of photosynthesis, although there is much less information for diatoms than for the Viridiplantae (Jensen et al., 2017). In the Viridiplantae, that includes the Embryophyta, carbon fixation by the Calvin-Benson-Bassham (CBB) cycle is well known to be fine-tuned by dark-light transitions, involving regulation by pH (Werdan et al., 1975), Mg^2+^ (Portis and Heldt, 1976), metabolite concentration (Anderson, 1973; Pupillo and Giulianipiccari, 1975; Gardemann et al., 1983; Baalmann et al., 1994), and primarily by the redox state of key enzymes (Buchanan et al., 1980; Schurmann and Jacquot, 2000). Non-covalently bound “small molecules” or metabolites also affect the rates of redox-interconversion of each redox-regulated enzyme in Viridiplantae and this fine-tuning regulation is well-described in a review from Knuesting and Scheibe (2018).

### Regulation by pH

In chloroplasts from Embryophyta, dark-to-light transitions are accompanied by a shift of the chloroplast internal pH from 7 in the dark to 8 in the light (Werdan and Heldt, 1973; Hauser et al., 1995). These changes directly regulate photosynthesis since many key chloroplastic enzymes have optimal activity at pH 8 and are much less active at pH 7 [reviewed in Gontero et al. (2007)]. In diatoms, pH responses have mainly been studied for external/environmental, rather than internal, pH. External pH can affect growth rate, silicon metabolism and biomineralization of *Conticribra weissflogii* (formerly known as *Thalassiosira weissflogii*) as well as its intracellular/cytoplasmic pH homeostasis (Herve et al., 2012). For other photosynthetic organisms, it is not the extracellular pH, but the intracellular pH in the chloroplast that is the critical factor for regulation of carbon acquisition, transport capacity and other metabolic processes. To our knowledge, internal pH has only been measured for a small number of diatoms. For *P. tricornutum* and *Cyclotella* sp. the pH was around 7 in the dark and 7.5 in the light (Colman and Rotatore, 1995), and for *Navicula pelliculosa*, it was 7.4 in the dark and 7.6 in the light (Colman and Rotatore, 1988). We found no published values for the pH within diatom chloroplasts stroma. One of the few studies of the effect of pH on enzyme activity in chromalveolates is for the chloroplastic glyceraldehyde-3-phosphate dehydrogenase (GAPDH) that catalyzes the reversible reduction and dephosphorylation of 1,3-bisphosphoglycerate to produce glyceraldehyde-3-phosphate and inorganic phosphate. Avilan et al. (Avilan et al., 2012) compared the optimal pH of GAPDH in the freshwater diatom, *Asterionella formosa*, the freshwater eustigmatophyte, *Pseudocharaciopsis ovalis*, and the model green alga, *C. reinhardtii*. In *A. formosa*, GAPDH was still active at the pH occurring in the dark, assuming that the internal pH (pH 7) reflects the one in the chloroplast. This suggests that GAPDH is regulated by factors other than pH in this diatom, unlike in the green algal enzyme that was down-regulated at the pH that occurs in the dark. The response of GAPDH from the eustigmatophyte *P. ovalis* was similar to that of the green alga *C. reinhardtii*. We do not know the internal chloroplast pH for *P. ovalis* but if the dark-to-light pH transition in this species is similar to that of *C. reinhardtii*, GAPDH could be partly regulated by pH under dark-light transitions. The different regulation of GAPDH by pH in the two heterokonts, *A. formosa* and *P. ovalis*, might be the result of the diverse evolutionary history of chromalveolates. Another example of regulation by pH is the lumenal enzyme violaxanthin de-epoxidase [VDE, (Lavaud et al., 2012)] that is involved in dissipating excess light energy (Lohr and Wilhelm, 1999).

Beyond photosynthesis, the carbon metabolism of the marine diatom *Skeletonema costatum* is regulated by the pH of the growth medium (Taraldsvik and Myklestad, 2000). The content of the carbohydrate storage compound, chrysolaminarin (β-1,3 linked glucan) decreased from 7.1 mg.L^−1^ at pH 6.5 to 0.2 mg.L^−1^ at pH 9.4 and concomitantly, the total organic carbon as glucan also decreased from 60 to 10%. The total amino acid content also decreased from 7.41 to 2.51 fmol.cell^−1^ when the pH of the growth medium increased (Taraldsvik and Myklestad, 2000). It is unclear if these are direct effects on carbon and nitrogen metabolism of external or internal pH or indirect effects linked to the greater external concentration of CO_2_ at pH 6.5 than at pH 9.4. Nevertheless, to relate these physiological responses to enzyme activity regulation, the authors report results from a Norwegian PhD thesis (Kirkvold, 1994) that showed that the specific activity of glutamine synthetase, a key enzyme in the metabolic pathway of glutamine and glutamate synthesis, also decreased with increasing pH when measured *in vitro*.

Studies on the effect of pH on activity should be expanded to more enzymes and their optimal pH compared to the internal pH in dark and light in order to determine if enzyme activity is regulated by internal pH. The difficulty of working with enzymes from diatoms and from chromalveolates in general, is probably responsible for the lack of data for this important group. For instance, in order to extract proteins from diatoms, litres of culture are required and it is not always easy to measure activity. Expressing recombinant diatom enzymes in heterologous systems is also challenging with many enzymes found in the insoluble fractions (B. Gontero, personal communication). Measurement of internal pH is also an experimental tour de force. Colman and Rotatore used the 5,5-dimethyl-2,4-oxazolidinadione distribution method that distributes between the medium and the cell as a function of their respective pH (Colman and Rotatore, 1988; Colman and Rotatore, 1995). However, this method does not distinguish between the pH in the cytoplasm, chloroplast stroma or thylakoid lumen.

### Regulation by the Redox State of Cysteine Residues

The redox control of enzyme activity in the Viridiplantae is primarily mediated by small proteins, thioredoxins, that are oxidized in the dark and reduced in the light (Buchanan et al., 1980; Buchanan, 2017). This regulation avoids futile cycles between the CBB and the OPP pathway, since both occur within the chloroplast, with enzymes from the CBB being active in the light and those from OPP being active in the dark. In contrast, in diatom plastids the OPP is incomplete, and presumably lacking (Kroth et al., 2008), and accordingly the regulation of their metabolism is different (Jensen et al., 2017). Moreover, diatoms have a high stromal reductant pressure and in contrast to Viridiplantae, metabolic activity in long dark periods leads to an enhanced reduction state of the plastoquinone pool. In the dark, since the plastoquinone pool is reduced, it may regulate redox-sensitive enzymes as is the case for algal nitrate reductase (Giordano et al., 2005). This avoids reducing equivalents to accumulate maintaining cellular redox poise (Wilhelm et al., 2006). In Viridiplantae in contrast, oxidizing conditions prevail in the dark, therefore suggesting that redox control may be different. Because of this unusual redox control, the redox regulation of diatom enzymes has been questioned (Wilhelm et al., 2006). However, diatoms possess many different thioredoxins, each encoded by a specific gene and located in different compartments, including the chloroplast. Most thioredoxins contain the regulatory cysteine residue in the conserved motif, WCGPC (Weber et al., 2009), thus they are likely to have specific regulation targets and some targets have been identified such as two CAs in *P. tricornutum* (Kikutani et al., 2012). The relatively few targets of thioredoxins currently identified in diatoms, contrasts with the 1188 targets found by combining qualitative and quantitative proteomic analyses in the *C. reinhardtii* thioredoxome (Perez-Perez et al., 2017).

**Figure 2:**
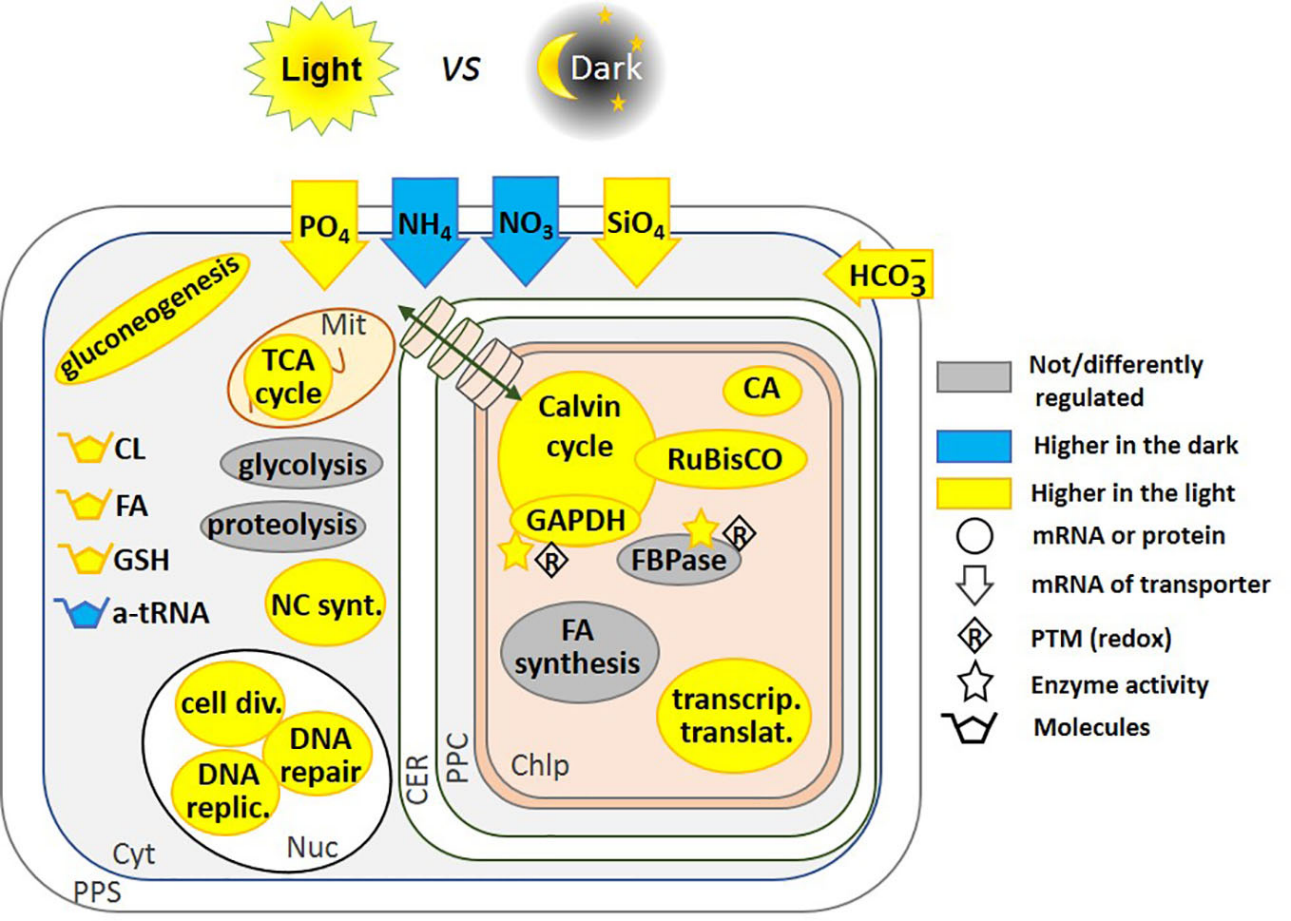
Regulation of pathways by light and dark. This schematic includes regulatory pathways from *P. tricornutum* (Chauton et al., 2013; Bai et al., 2016), and *T. pseudonana* (Ashworth et al., 2013). Where up-regulation of the mRNA transcription preceded the start of the photoperiod, we have represented it as being higher in the light, this is the case for RuBisCO expression in *T. pseudonana* (Ashworth et al., 2013). Glycolysis and proteolysis pathways are up-regulated in the light in *T. pseudonana*, but down-regulated in *P. tricornutum*. In contrast, fatty acid synthesis is down-regulated in *T. pseudonana* but up-regulated in *P. tricornutum*. Fatty acid content increased after 3 h of light in *P. tricornutum* (Chauton et al., 2013), we have thus represented the molecule as being higher in the light. FBPase gene expression is not regulated, but its activity is redox-regulated (Michels et al., 2005; Mekhalfi et al., 2012). Redox regulation of enzyme activity has been shown on the isolated proteins *in vitro*, and by analogy with the Viridiplantae lineage, this could be related to light-to-dark transitions. The transport of molecules between the cytoplasm and the chloroplast is represented by a green double-headed arrow across cylinders. CL, Chrysolaminarin; FA, Fatty Acid; GSH, Glutathione; a-tRNA, aminoacyl-transfer RNA; CA, Carbonic Anhydrase; NC synt., Nucleotide synthesis; cell div., Cell division; DNA replic., DNA replication; PPS, Periplasmic Space; CER, Chloroplast Endoplasmic Reticulum; PPC, Periplastidial Compartment; Mit, Mitochondrion; Chlp, Chloroplast; Cyt, Cytoplasm; Nuc, Nucleus.

Using a redox proteomics approach on *P. tricornutum*, Rosenwasser et al. identified the “redoxome”, or in other words the redox-sensitive proteins, and demonstrated its involvement in photosynthesis, photorespiration, lipid biosynthesis, and nitrogen metabolism (Rosenwasser et al., 2014). In that case, however, the redox-sensitivity is a response to oxidative stress rather than light-dark transition even though reactive oxygen species are photo-induced, and increase in parallel to glutathione (GSH). GSH is a low-molecular-weight tripeptide that consists of cysteine (Cys), glutamic acid (Glu), and glycine (Gly) and is present in microorganisms, plants, and mammals (Zaffagnini et al., 2012). It can regulate protein activity by forming a mixed disulfide bridge between the thiol group of its Cys and an accessible free thiol on a protein, a process known as protein *S*-glutathionylation (Zaffagnini et al., 2012; Marri et al., 2014). This post-translational modification can protect specific Cys residues from irreversible oxidation but can also modulate protein activities (Zaffagnini et al., 2012; Marri et al., 2014; Thieulin-Pardo et al., 2015). In *T. pseudonana*, a diurnal redox-related pattern has been observed in which GSH accumulates in the light, and decreases upon darkness, (Dupont et al., 2004) (**Figure 2**). However, direct regulation of the enzyme activities by glutathionylation in diatoms, or in other chromalveolates, in contrast to Viridiplantae, has not yet been studied, to the best of our knowledge.

Although the effect of glutathionylation on enzymes has not been studied in diatoms, the effect of other reducing agents such as dithiothreitol has been investigated, though understudied as compared to other photosynthetic organisms such as Cyanobacteria, Chlorophyta, Rhodophyta, and Embryophyta. The chloroplastic phosphoglycerate kinase belonging to the CBB cycle, catalyzes the ATP-Mg^2+^-dependent phosphorylation of 3-phosphoglycerate (3-PGA) to 1,3-bisphosphoglycerate, in a reversible reaction and was redox-regulated in *P. tricornutum* (Belen Bosco et al., 2012). However, in our hands, PGK was not redox-regulated, as was also the case in *T. pseudonana*, in *Navicula pelliculosa* grown with sea water and fresh water medium and in a freshwater diatom, *A. formosa* (Jensen et al., 2019b). In contrast to the Viridiplantae, two enzymes that are unique to the CBB were not redox regulated (Michels et al., 2005; Maberly et al., 2010; Jensen et al., 2017). These include, sedoheptulose 1,7-bisphosphatase that irreversibly catalyzes the dephosphorylation of sedoheptulose-1,7-bisphosphate producing sedoheptulose-7-phosphate, and phosphoribulokinase (PRK) that irreversibly catalyzes the ATP-Mg^2+^-dependent phosphorylation of ribulose-5-phosphate into ribulose-1,5-phosphate. The general lack or weak redox regulation of PRK in the chromalveolates (diatoms and other groups) seems to be related to its sequence, where the connectivity between two regulatory cysteine residues is crucial [at position 16 and 55 in *C. reinhardtii* (Maberly et al., 2010)]. In many photosynthetic organisms PRK can also be sequestered, and inactivated, in a PRK-GAPDH-CP12 complex, that has not yet been found in diatoms. The absence of the ternary complex with GAPDH in diatoms has been attributed to the absence of two cysteine residues on PRK (at position 243 and 249 numbered from the enzyme from *C. reinhardtii*) that are present in Cyanobacteria, Chlorophyta, Rhodophyta, and Embryophyta where the complex has been identified (Thieulin-Pardo et al., 2015). In contrast however, a ferredoxin-NADP reductase (FNR)-GAPDH-CP12 complex has been found in *A. formosa* (Mekhalfi et al., 2014). For the chloroplastic GAPDH, the regulation is more complex as discussed above in the pH regulation section, but in many diatoms, this enzyme seems to be redox regulated (Maberly et al., 2010; Mekhalfi et al., 2012; Mekhalfi et al., 2014; Jensen et al., 2019b).

### Direct Light-Dark Control of Gene Expression

Regulation at the transcriptional level by light-dark transitions, occurs in Viridiplantae, and also in diatoms (Sun et al., 2003; Fey et al., 2005). In *T. pseudonana*, after 12 h of light, 1,859 genes were upregulated compared to cells exposed to 12 hours of dark, and inversely, after 12h of dark, 1,326 genes were up-regulated compared to cells exposed to 12 h of light (Ashworth et al., 2013). Among the most highly expressed genes after 12 h of light were the ones encoding enzymes for cell division, DNA replication and repair, carbon metabolism and oxidative phosphorylation while after 12 h of dark, the most highly expressed genes were those encoding ribosomal biogenesis, aminoacyl-tRNA and key photosynthetic enzymes (**Figure 2**). Some genes, such as that encoding RuBisCO, anticipates the diurnal cycle and is up-regulated before the onset of light. The dark-light expression pattern of genes was affected by growth phase (exponential *vs.* stationary). In the stationary phase, the expression of only a few genes fluctuated under dark-light transitions (Ashworth et al., 2013). One of these genes encodes a putative pyruvate carboxylase suggesting a switch toward other types of metabolism such as gluconeogenesis and lipid biosynthesis. This might be explained by the hypothesis raised by Norici et al. in *S. marinoi* (Norici et al., 2011) who postulated that the diatom re-routes its metabolism toward lipid biosynthesis, because of the relatively high volume-based energy content of lipids in an organism in which size decreases with vegetative cell divisions, thus requiring carbon allocation into more energy-compact compounds.

In the light, more than 4,500 transcripts were differentially expressed in *P. tricornutum*, including genes such as the one encoding for pyruvate transporter that had never been previously described in this organism (Chauton et al., 2013). This work shows that transcriptional regulation of carbohydrate and lipid metabolism occurs in diatoms (**Figure 2**). Indeed, the content of soluble glucans and lipids decreased in the dark and fatty acid biosynthesis genes were up-regulated within 30 min of a switch from dark to light. Fatty acid biosynthesis and the tricarboxylic acid (TCA) cycle are also tightly co-ordinated (Chauton et al., 2013). During the day, carbon skeletons are produced within the chloroplast while in the night these carbon-rich compounds are broken down in the mitochondria and the cytosol.

Interestingly, four carbon fixation enzymes were co-regulated in *P. tricornutum:* PGK, GAPDH, triose phosphate isomerase/GAPDH and PRK. Their mRNAs were all highest at the beginning of the light period (dawn) and lowest at the beginning of the dark period (dusk). Bai et al. (2016) showed however that the expression of PRK increased after 4 days of dark treatment using a proteomic approach. Similarly, the expression of phosphoribose isomerase, the enzyme that produces PRK substrate, as well as the expression of transaldolase and glyoxylase, increased after dark treatment.

Redox regulation of PRK is observed and well-studied in Viridiplantae, but in contrast in diatoms and other chromalveolates, as mentioned above, the activity of PRK was affected neither by oxidizing nor by reducing conditions (Maberly et al., 2010). Therefore, for enzymes that are not redox-regulated, regulation at the transcriptional level could allow a dark-light regulation of the CBB. The expression pattern of the gene of fructose-1,6-bisphosphatase (FBPase) that catalyzes the dephosphorylation of fructose-1,6-bisphosphate into fructose-6-phosphate and inorganic phosphate, was not induced to the same degree by light as other CBB enzymes. Since this enzyme can be redox regulated (Michels et al., 2005; Mekhalfi et al., 2012), its regulation could occur both at the level of activity and transcription.

Carbon acquisition is also stopped at night since in *P. tricornutum*, mRNA encoding for bicarbonate transporters of the Solute Carrier family 4 (SLC4) and for alpha-carbonic anhydrases, especially α-CA-VII, were much less abundant in the dark than in the light (Chauton et al., 2013). Recently, in the same organism, the pattern of mRNA levels at a photon irradiance of 30,300, 1,000 µmol photon m^−2^ s^−1^ differed at the lag, exponential and stationary phases of growth (Heydarizadeh et al., 2019).

Dark-treated *P. tricornutum* cells preferentially utilize carbon and nitrogen obtained from protein breakdown to increase lipid cell quotas at low cost (Bai et al., 2016). Long-term dark stress inhibited several key proteins involved in nitrogen assimilation and in the synthesis of the photosynthetic machinery. Simultaneously, key enzymes of glycolysis and the synthesis of fatty acids were induced apparently to assimilate the excess of C and N from protein breakdown. Uptake of other resources for growth are also light-regulated: transporters for uptake of phosphate and silica are higher in the light and nitrate and ammonium transporters are higher in the dark (Ashworth et al., 2013) (**Figure 2**). Data from the literature showing differential expression of proteins, transcripts and metabolites in light and dark are summarized in **Figure 2**.

### Enzyme Activity, Metabolite Concentration, and Carbon Storage Compounds

Gene expression gives important clues on how light (quality, irradiance, duration) affects metabolism. However, it is also necessary to measure enzyme activity and metabolite concentration as these are the ultimate response to environmental change. For instance, an excess of light modifies lipid biosynthesis in the coastal marine diatom, *Skeletonema marinoi* (Norici et al., 2011). In *S. costatum*, carbohydrate increased with irradiance (Hitchcock, 1980) while lipids increased in *Chaetoceros calcitrans* (Harrison et al., 1990) and therefore the carbon allocation seems to be different and species-specific. Under different light-regimes, different species behave differently and the amount of essential fatty acids with growth irradiance is also species-specific. These examples illustrate that although enzyme activities have been measured, the mechanism underlying the change in activity is unknown. For example, PEPCK that converts oxaloacetate into PEP and CO_2_ in gluconeogenesis, increased 2.5-fold in cells of *S. marinoi* grown under low light (25 µmol.photon.m^−2^.s^−1^) *vs.* high light (250 µmol.photon.m^−2^.s^−1^). The authors suggested that this enzyme might be involved in the conversion of lipid to carbohydrates especially under low light (Norici et al., 2011). In contrast, since energy demand for lipid synthesis is much higher than for carbohydrate synthesis (Raven et al., 2005; Subramanian et al., 2013) under excess light, lipids represent a better sink for excess energy. As a consequence, lipid accumulation in high irradiance was observed in *S. marinoi*, although this is not always the case. In this species, other enzymes are probably not regulated by light since their activity remains unchanged. For instance, the activity of PEPC, that catalyzes the addition of bicarbonate (HCO_3_^−^) to PEP to produce oxaloacetate, was similar for cells grown at low or high light. This enzyme is involved in C4 metabolism and in anaplerotic reactions. Similarly, the activity of glutamine synthetase that is involved in photorespiration did not change in cells grown at low or high light.

## Effect of CO_2_

Like other algae, diatoms exhibit a range of responses to varying CO_2_ concentration, including effects on photophysiology, rate of photosynthesis and growth, chemical and pigment composition, and community species composition, but there are large species- and context-specific variations in the magnitude and sign of response (Boelen et al., 2011; Torstensson et al., 2012; Gao and Campbell, 2014; Dutkiewicz et al., 2015; Bach and Taucher, 2019; Jensen et al., 2019b). At the ocean surface, the air-equilibrium concentration of CO_2_ (Dickson, 2010) varies between 5 and 25 µM depending on temperature (Raven, 1994; Tortell, 2000; Kim et al., 2006; Matsuda et al., 2011; Maberly and Gontero, 2017). This CO_2_ concentration is insufficient to saturate the carboxylating enzyme, RuBisCO (Young et al., 2016) and may not saturate rates of diatom growth or photosynthesis (Riebesell et al., 1993; Dutkiewicz et al., 2015). This is mitigated by CO_2_-concentrating mechanisms (CCMs) (Hopkinson et al., 2011) that are facultative and increase the concentration of CO_2_ around RuBisCO, and are present in many phytoplankton. CCMs can involve biophysical or biochemical processes (Reinfelder et al., 2000; Reinfelder, 2011; Hopkinson et al., 2016) although the latter is controversial in diatoms (Clement et al., 2017a). The CCM regulation in diatoms is highly dependent on light as well as CO_2_ concentration (Harada et al., 2006). However, the major determinant of the extent of CCM expression in *P. tricornutum* is CO_2_ concentration, as it is in green algae (Matsuda and Kroth, 2014) and many components of CCM are suppressed under elevated CO_2_ concentrations and induced at atmospheric levels or lower. The CO_2_ concentration affects expression, and consequently the activity, of not only CCMs components but also that of enzymes from metabolic pathways such as the CBB cycle and glycolysis, though this is still understudied. Below, we compile information on the regulation by CO_2_ at the transcriptional and/or the enzyme activity levels of enzymes involved in the CCM and other metabolic pathways.

### CO_2_-Concentrating Mechanisms (CCMs)

#### Biochemical CCM

**Figure 3:**
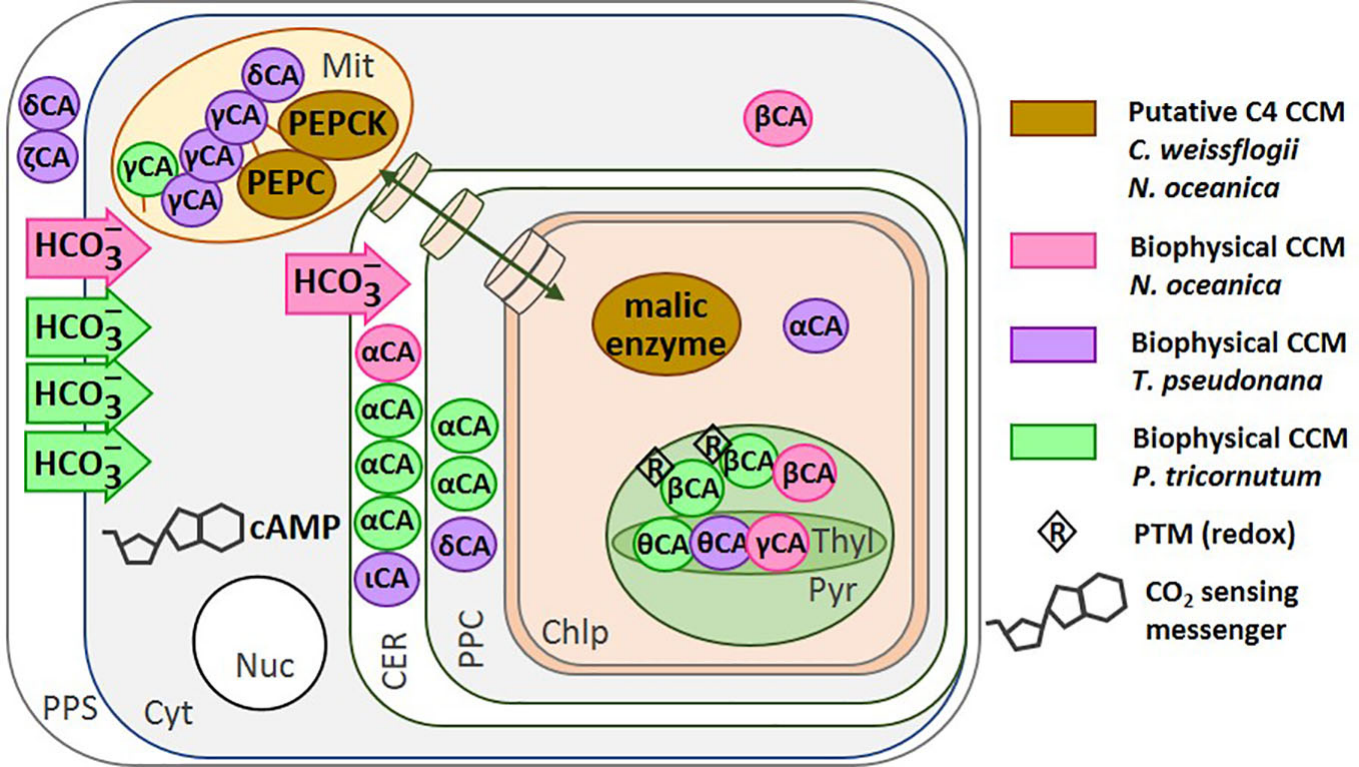
Location of the CCM components in different diatom species. The carbonic anhydrases (circles) and bicarbonate transporters (arrows) are shown for: *N. oceanica* (Wei et al., 2019) (magenta), *T. pseudonana* (Samukawa et al., 2014; Jensen et al., 2020) (purple), and *P. tricornutum* (Jensen et al., 2020) (green). The putative C4-CCM components proposed for *N. oceanica* (Wei et al., 2019) and *C. weissflogii* (Reinfelder et al., 2000; Ohno et al., 2012; Hopkinson et al., 2016; Tanaka et al., 2016). The transport of molecules between the cytoplasm and the chloroplast is represented by a green double-headed arrow across cylinders. Redox regulation of CA activity has been shown on the isolated *P. tricornutum* β-CA *in vitro*. PEPC, Phosphoenolpyruvate Carboxylase; PEPCK, Phosphoenolpyruvate Carboxykinase; CA, Carbonic Anhydrase; PPS, Periplasmic Space; CER, Chloroplast Endoplasmic Reticulum; PPC, Periplastidial Compartment; Mit, Mitochondrion; Chlp, Chloroplast; Cyt, Cytoplasm; Nuc, Nucleus. Figure adapted from Jensen et al. (2020).

In C4 metabolism, PEPC is the first carboxylating enzyme and traps bicarbonate into a C4 carbon compound. This compound is subsequently cleaved by a decarboxylase enzyme to provide a 3-carbon compound and CO_2_, near the active site of RuBisCO (Sage, 2004). As mentioned above, the presence of C4 or biochemical CCM in diatoms does not seem to be universal. For instance, there is evidence for it in *C. weissflogii* (Reinfelder et al., 2000; Reinfelder, 2011; Hopkinson et al., 2016) but it is absent in *P. tricornutum* (Haimovich-Dayan et al., 2013; Clement et al., 2017a; Ewe et al., 2018). In the eustigmatophyte *Nannochloropsis oceanica*, a novel type of C4-based CCM was proposed to occur when cells were shifted from high CO_2_ (50,000 ppm) to low CO_2_ (100 ppm) (Wei et al., 2019). In this C4-based CCM, PEPC and PEPCK have been proposed to be involved in the primary inorganic carbon fixation steps in mitochondria, and not in chloroplasts. Subsequent decarboxylation of malate by a malic enzyme in the chloroplast enriches CO_2_ in the vicinity of RuBisCO (**Figure 3**). Transcripts levels of some C4 enzymes were not altered by CO_2_ concentration and do not suggest a classic C4 metabolism, but activities of PEPC and malic enzyme increased under low CO_2_ (Wei et al., 2019). More work is required to confirm this interesting possibility.

#### Biophysical CCM

In the genomes of *P. tricornutum* and *T. pseudonana*, nine and thirteen CA gene sequences have been found respectively (Tachibana et al., 2011; Samukawa et al., 2014). In *P. tricornutum*, the two chloroplastic pyrenoidal β-CAs PtCA1 and 2, responded to CO_2_ (Satoh et al., 2001; Harada and Matsuda, 2005; Harada et al., 2005) and later this was confirmed by Tachibana et al. by semi-quantitative RT-PCR (Tachibana et al., 2011). The activation of both PtCA1 and PtCA2 under CO_2_ limitation involves three *cis*-regulatory elements, TGACGT, ACGTCA, and TGACGC, at a region minus 86 to minus 42 upstream of the transcription start site. These elements, CCRE1 to 3, are critical for the transcriptional response to ambient CO_2_ *via* the level of the second messenger cAMP (Ohno et al., 2012; Tanaka et al., 2016). The sensing of CO_2_ mediated by cAMP has been reported in cyanobacteria, fungi and mammals (Matsuda et al., 2011) and also in *T. pseudonana* (Hennon et al., 2015; Young and Morel, 2015). The transcriptional activation of PtCA2 in response to the decrease in CO_2_ concentration was strongly light-dependent, such that either CO_2_ or the absence of light can down-regulate the promoter. In contrast, CO_2_ concentration and light have additive effects on the regulation of PtCA1 (Tanaka et al., 2016). It is worth remarking that both PtCA1 and PtCA2 were post translationally regulated by redox modifications *via* thioredoxins (Kikutani et al., 2012) (**Figure 3**).

Tachibana et al. also showed that three putative CA genes in *T. pseudonana*, CA-1, 3, and 7 (α-CA, ζ-CA, and δ-CA, respectively) were induced by decreasing CO_2_, and function in CO_2_-limited environments (Tachibana et al., 2011). Similarly, in *C. weissﬂogii*, both CO_2_ and HCO_3_^−^ uptake increased in response to a CO_2_ decrease and this was accompanied by an increase in both internal and external CA activity (Burkhardt et al., 2001). A recent proteomic study on *T. pseudonana* acclimated to low CO_2_ (50 ppm) revealed a new uncharacterized protein, later called LCIP63, for “low-CO_2_-inducible protein of 63 kDa” that was up-regulated under low CO_2_ (50 ppm) or at atmospheric CO_2_ (400 ppm) (Clement et al., 2017a) but down-regulated when nitrogen, phosphorus or silicon were limiting conditions (Lin et al., 2017; Chen et al., 2018). LCIP63 was up-regulated in *T. pseudonana* growing at 300 ppm *vs.* 1,000 ppm CO_2_ (Valenzuela et al., 2018). Recently, this protein was identified as a new CA [designed as iota CA, (Jensen et al., 2019a)] that uses Mn^2+^ as a co-factor instead of the more common divalent cation Zn^2+^ (Tsuji et al., 2017; Dimario et al., 2018). The gene of iota CA is also present in bacterial genomes (Jensen et al., 2019a) and recently, the gene encoding this enzyme from the gram negative bacterium *Burkholderia territorii* was cloned and the purified recombinant protein showed a CA activity using Zn^2+^ instead of Mn^2+^ (Del Prete et al., 2020) indicating that the use of Mn^2+^ as a co-factor could be a feature of diatoms.

In *N. oceanica*, transcriptomic, proteomic and metabolomic data are available for cells grown at high CO_2_ (50,000 ppm) and low CO_2_ (100 ppm); three of the CA transcripts were up-regulated (β-CA-2, β-CA-4 and α-CA-5) under low CO_2_ (Wei et al., 2019). In addition, the transcripts of bicarbonate transporters of the SLC4 family and several of the ABC transporter family were also more abundant at low CO_2_, indicating an active biophysical CCM in this organism. Similarly, in *P. tricornutum*, beside the regulation of numerous CAs by CO_2_, three out of seven SLC4 genes were induced by low CO_2_ and were highly inhibited by the anion exchange inhibitor 4,4'-diisothiocyanatostilbene 2,2'-disulphonate (Nakajima et al., 2013). In *T. pseudonana*, genes homologous to those in *P. tricornutum* have been found (Matsuda et al., 2017); however, their functionality at the protein level has not yet been studied. Two chloroplast transporters of the bestrophin family of anion channels that are permeable to HCO_3_^−^ were also more abundant at low CO_2_ and may play a role in the biophysical CCM of this diatom (Kustka et al., 2014). These data are summarized in **Figure 3**.

Depending on future CO_2_ emission scenarios, atmospheric levels of CO_2_ are likely to reach 800 ppm by 2,100 (IPCC, 2014; Gattuso et al., 2015). This relates to a temperature-dependent dissolved CO_2_ concentration at the ocean surface of 25 to 50 μM. At this concentration, cAMP plays a crucial role in down-regulating CCM genes in *T. pseudonana*, in particular those encoding the chloroplastic δCA3, some transporters and some involved in photorespiration (e.g., glycolate oxidase). These photorespiration genes and CCM genes interestingly, belong to a single CO_2_-responsive cluster that shares the same upstream *cis*-regulatory sequences found in *P. tricornutum* that is also responsible for down-regulation of the β-CA gene upon increased CO_2_ (Ohno et al., 2012). Similarly, genes involved in photosynthesis, the TCA cycle, oxidative phosphorylation and protein degradation were down-regulated, while in contrast other genes involved in signalling mechanisms were up-regulated at 800 ppm compared to 400 ppm CO_2_ (Hennon et al., 2015). Not all these genes contain the upstream regulatory region, though they were highly affected. This regulation is likely an indirect effect linked to the impact of high CO_2_ on the genes of structural maintenance of chromosomes (SMC), transcription factors, and histones.

Sensing a change, either an increase or a decrease, in external CO_2_ concentrations through cAMP seems to be a general rule of gene regulation rather than an exception even in diatoms (Young and Morel, 2015).

### Enzymes From Carbon Metabolism

**Figure 4:**
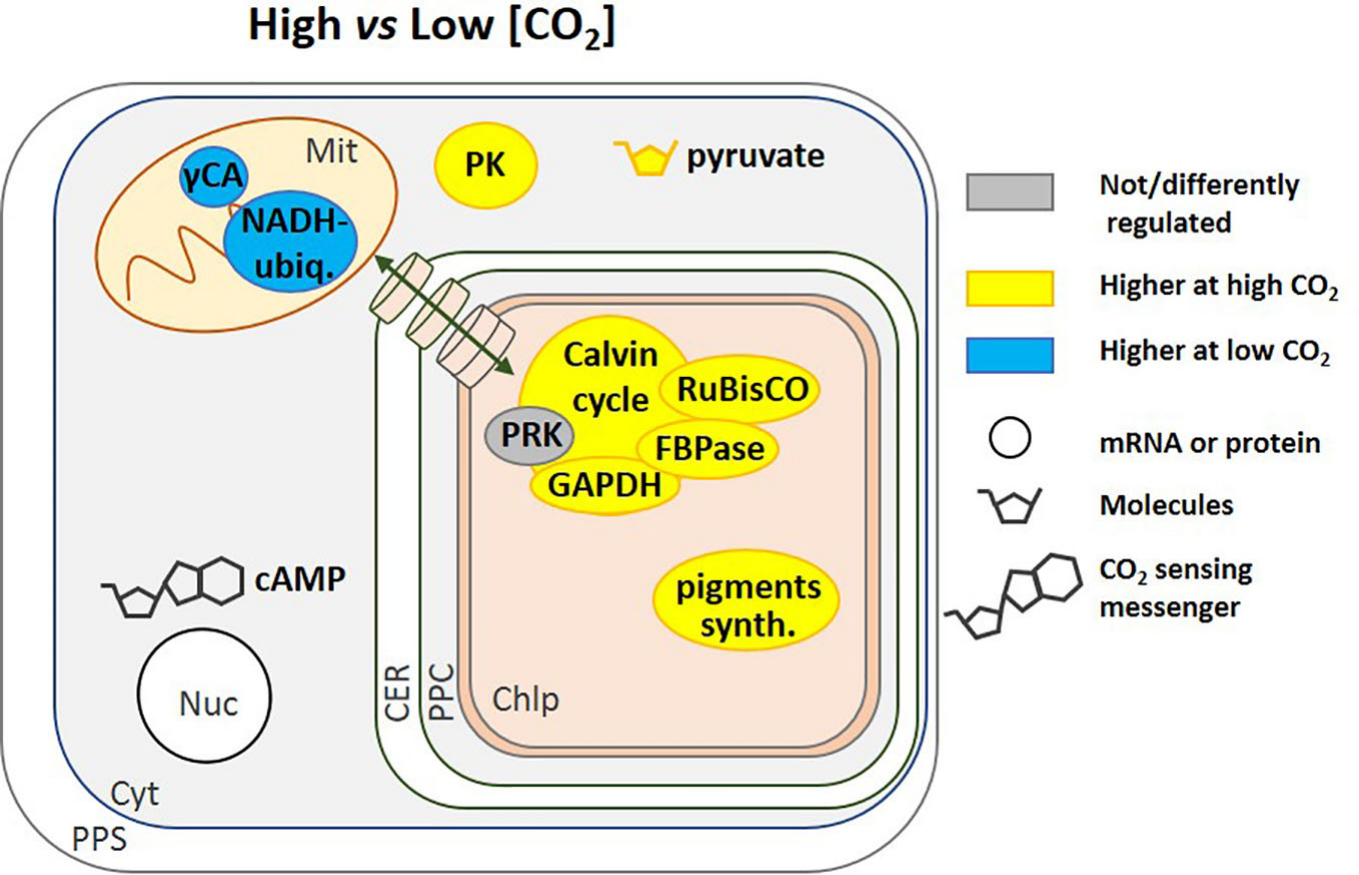
Regulation of pathways by [CO_2_]. This schematic includes regulatory pathways from different species, including *N. oceanica* (Wei et al., 2019) and *T. pseudonana* (Clement et al., 2017b; Jensen et al., 2019b). Cyclic AMP (cAMP) is a general [CO_2_] signalling molecule that regulates gene expression (Ohno et al., 2012; Hennon et al., 2015; Young and Morel, 2015). High CO_2_ corresponds to 20,000 to 50,000 ppm but in Hennon et al. (2015) it is 800 ppm and low CO_2_ is 50 to 400 ppm. The transport of molecules between the cytoplasm and the chloroplast is represented by a green double-headed arrow across cylinders. NADH ubiq., NADH-ubiquinone oxidoreductase complex; PK, Pyruvate Kinase; pigments synth., pigments synthesis; CA, Carbonic Anhydrase; PPS, Periplasmic Space; CER, Chloroplast Endoplasmic Reticulum; PPC, Periplastidial Compartment; Mit, Mitochondrion; Chlp, Chloroplast; Cyt, Cytoplasm; Nuc, Nucleus.

Many related metabolic pathways are affected by CO_2_ in addition to CCM expression. Gamma CA and the NADH-ubiquinone oxidoreductase complex (C1) are associated in many organisms (Cardol, 2011) and in *N. oceanica*, at very low [CO_2_] (100 ppm), the genes coding for these two mitochondrial enzymes were up-regulated (**Figure 4**). Since they can facilitate transport of CO_2_ produced by the TCA cycle and photorespiration toward the chloroplast, in the form of bicarbonate, they contribute to what is called a basal CCM (Wei et al., 2019). At the enzyme activity level, *T. pseudonana* cells grown at high CO_2_ concentration (20,000 ppm) *vs.* atmospheric (400 ppm) displayed higher NADPH-dependent GAPDH and FBPase activity (**Figure 4**) indicating that the CBB cycle could be affected even though PRK activity was unaltered (Clement et al., 2017b). The activity of pyruvate kinase, a glycolytic enzyme, was also strongly stimulated when cells were grown at 20,000 ppm (**Figure 4**) while two other glycolytic enzymes, NADH-dependent GAPDH and glucose-6-phosphate dehydrogenase (G6PDH) were unaffected (Clement et al., 2017b). In this diatom, a model summarizing the effect of inorganic carbon limitation, based both on activities and protein expression profiles has been elaborated (Clement et al., 2017b). This model shows the remodelling of metabolism with a diversion of energy and resources toward carbon metabolism at high CO_2_ and toward carbon capture at low CO_2_. An increase in δ-CA gene expression, to capture as much CO_2_ as possible, was also observed by Kutska et al. (2014) in *T. pseudonana*. Metabolism enzymes, as well as enzymes responsible for pigment synthesis and indirectly in light capture (**Figure 4**), were less abundant, while enzymes involved in reactive oxygen species (ROS) defence increased in order to avoid accumulation of ROS that might occur when energy is in excess. The increased activity of pyruvate kinase (**Figure 4**), observed in *T. pseudonana* seems to be shared by other diatoms, as it was observed in many diatoms from freshwater to seawater species grown under high CO_2_ (20,000 ppm) *vs.* atmospheric CO_2_ (400 ppm) (Jensen et al., 2019b). In addition, a modification of gene expression has been described that allows synthesis of either PEP or pyruvate under carbon shortage, indicating that pyruvate is an important hub in these organisms (Heydarizadeh et al., 2017; Heydarizadeh et al., 2019) (**Figure 4**).

## Conclusions and Future Directions

Physiological and genomic data are available for the response of some chromalveolates, especially diatoms, to light and CO_2_. They reveal the multitude and complexity of mechanisms that these organisms have evolved to cope with environmental variation. However, chromalveolates are still understudied compared to the Viridiplantae and more research is needed to unravel fully how this important group of algae maintain their productivity under changing conditions. There is a particular lack of information in diatoms on internal pH, especially in the chloroplast, on the identity of redox-regulated enzymes, on regulation by post-translational modification and on protein-protein interactions. There are existing and new methods that could be employed to tackle these knowledge gaps. For example, a range of pH-sensitive fluorescent probes are available to measure internal pH (Loiselle and Casey, 2010), although their low ability to penetrate the cell and organelle can be challenging. However, internal pH has also been measured successfully using the inorganic phosphate (^31^P) nuclear magnetic resonance frequency in other organisms such as fungi (Hesse et al., 2000) and the anammox bacterium, *Kuenenia stuttgartiensis* (Van Der Star et al., 2010), and this could be applied to chromalveolates.

There are a growing number of studies taking advantage of proteomics to study diatom responses to stress, e.g. Muhseen et al. (2015) or Chen et al. (2018). Proteomics approaches have been used successfully in Viridiplantae to identify candidates for thioredoxin interactions (Montrichard et al., 2009; Perez-Perez et al., 2017). This could be extended to diatoms where there is a real challenge to assign specific targets to each of the many thioredoxins found in diatoms (Weber et al., 2009). Biotin-based proximity labelling approaches, coupled to quantitative proteomics, such as APEX BioID, are emerging tools for the study of protein-protein interactions (Santin et al., 2018; Beganton et al., 2019) that could be developed for chromalveolates. There is evidence for unusual PTMs involved in the regulation of RuBisCO from an arctic diatom (Valegard et al., 2018) and proteomics could also be a powerful approach to analyse these modifications. For instance, a phosphoproteomic analysis in *P. tricornutum* confirmed that phosphorylation occurs in many metabolic pathways (Chen et al., 2014).

Over-expressing or silencing a gene is starting to be applied to diatoms in order to determine their metabolic role (Hildebrand et al., 2017; Huang and Daboussi, 2017; Falciatore et al., 2020). Recently, tailored TALEN endonucleases and the CRISPR/Cas9 system have been utilized in diatoms (Daboussi et al., 2014; Hopes et al., 2016; Nymark et al., 2016), allowing knockout strains with targeted genetic modifications to be produced. An overview of the genetic toolbox currently available for performing stable genetic modifications in diatoms is reviewed in Kroth et al. and Falciatore et al. (Kroth et al., 2018; Falciatore et al., 2020).

It is clear that the techniques mentioned above in combination with genome sequencing, “omics” and targeted approaches, will allow the biology of diatoms and chromalveolates to be understood better. However, since many responses seem to be species-specific, a wider range of species need to be studied, especially those from non-marine systems, to produce a more complete picture of the functioning in this supergroup with a mosaic of multi-lineage genomes.

## Author Contributions

All authors contributed to the article and approved the submitted version.

## Funding

CNRS and Aix Marseille are our current funders and the ANR-15-CE05-0021-03 allows us to work on diatom project.

## Conflict of Interest

The authors declare that the research was conducted in the absence of any commercial or financial relationships that could be construed as a potential conflict of interest.

## References

[B1] Alissa S. A. Alghulikah H. A. Alothman Z. A. Osman S. M. Del Prete S. Capasso C. (2020). Inhibition survey with phenolic compounds against the delta- and eta-class carbonic anhydrases from the marine diatom *thalassiosira weissflogii* and protozoan *Plasmodium falciparum*. J. Enzym. Inhib. Med. Ch. 35, 377–382. 10.1080/14756366.2019.1706089 PMC6968676 31856608

[B2] Allen A. E. Dupont C. L. Obornik M. Horak A. Nunes-Nesi A. Mccrow J. P. (2011). Evolution and metabolic significance of the urea cycle in photosynthetic diatoms. Nature 473, 203–207. 10.1038/nature10074 21562560

[B3] Anderson L. E. (1973). Regulation of pea leaf ribulose-5-phosphate kinase-activity. Biochim. Biophys. Acta 321, 484–488. 10.1016/0005-2744(73)90190-3 4357662

[B4] Armbrust E. V. Berges J. A. Bowler C. Green B. R. Martinez D. Putnam N. H. (2004). The genome of the diatom *Thalassiosira pseudonana*: Ecology, evolution, and metabolism. Science 306, 79–86. 10.1126/science.1101156 15459382

[B5] Ashworth J. Coesel S. Lee A. Armbrust E. V. Orellana M. V. Baliga N. S. (2013). Genome-wide diel growth state transitions in the diatom *Thalassiosira pseudonana*. Proc. Natl. Acad. Sci. U.S.A. 110, 7518–7523. 10.1073/pnas.1300962110 23596211 PMC3645528

[B6] Avilan L. Maberly S. C. Mekhalfi M. Plateau J. Puppo C. Gontero B. (2012). Regulation of glyceraldehyde-3-phosphate dehydrogenase in the eustigmatophyte *Pseudocharaciopsis ovalis* is intermediate between a chlorophyte and a diatom. Eur. J. Phycol. 47, 207–215. 10.1080/09670262.2012.687459

[B7] Baalmann E. Backhausen J. E. Kitzmann C. Scheibe R. (1994). Regulation of NADP-dependent glyceraldehyde-3-phosphate dehydrogenase-activity in spinach-chloroplasts. Bot. Acta 107, 313–320. 10.1111/j.1438-8677.1994.tb00801.x

[B8] Bach L. T. Taucher J. (2019). CO_2_ effects on diatoms: a synthesis of more than a decade of ocean acidification experiments with natural communities. Ocean Sci. 15, 1159–1175. 10.5194/os-15-1159-2019

[B9] Bai X. Song H. Lavoie M. Zhu K. Su Y. Ye H. (2016). Proteomic analyses bring new insights into the effect of a dark stress on lipid biosynthesis in *Phaeodactylum tricornutum*. Sci. Rep. 6, 25494. 10.1038/srep25494 27147218 PMC4857112

[B10] Bailleul B. Berne N. Murik O. Petroutsos D. Prihoda J. Tanaka A. (2015). Energetic coupling between plastids and mitochondria drives CO_2_ assimilation in diatoms. Nature 524, 366–369. 10.1038/nature14599 26168400

[B11] Basu S. Patil S. Mapleson D. Russo M. T. Vitale L. Fevola C. (2017). Finding a partner in the ocean: molecular and evolutionary bases of the response to sexual cues in a planktonic diatom. New Phytol. 215, 140–156. 10.1111/nph.14557 28429538 PMC5485032

[B12] Beganton B. Coyaud E. Mange A. Solassol J. (2019). New approaches for protein-protein interaction study. Med. Sci. 35, 223–231. 10.1051/medsci/2019035 30931906

[B13] Belen Bosco M. Cristina Aleanzi M. Alvaro Iglesias A. (2012). Plastidic phosphoglycerate kinase from *Phaeodactylum tricornutum*: on the critical role of cysteine residues for the enzyme function. Protist 163, 188–203. 10.1016/j.protis.2011.07.001 21816671

[B14] Boelen P. De Poll W. H. V. Van Der Strate H. J. Neven I. A. Beardall J. Buma A. G. J. (2011). Neither elevated nor reduced CO_2_ affects the photophysiological performance of the marine Antarctic diatom *Chaetoceros brevis*. J. Exp. Mar. Biol. Ecol. 406, 38–45. 10.1016/j.jembe.2011.06.012

[B15] Bowler C. Allen A. E. Badger J. H. Grimwood J. Jabbari K. Kuo A. (2008). The *Phaeodactylum* genome reveals the evolutionary history of diatom genomes. Nature 456, 239–244. 10.1038/nature07410 18923393

[B16] Brembu T. Winge P. Tooming-Klunderud A. Nederbragt A. J. Jakobsen K. S. Bones A. M. (2014). The chloroplast genome of the diatom *Seminavis robusta*: new features introduced through multiple mechanisms of horizontal gene transfer. Mar. Genom. 16, 17–27. 10.1016/j.margen.2013.12.002 24365712

[B17] Buchanan B. B. (1980). Role of light in the regulation of chloroplast nzymes. Ann. Rev. Plant Physiol. 31, 341–374. 10.1146/annurev.pp.31.060180.002013

[B18] Buchanan B. B. (2017). The path to thioredoxin and redox regulation beyond chloroplasts. Plant Cell Physiol. 58, 1826–1832. 10.1093/pcp/pcx119 29016988

[B19] Burkhardt S. Amoroso G. Riebesell U. Sultemeyer D. (2001). CO_2_ and HCO_3_^-^ uptake in marine diatoms acclimated to different CO_2_ concentrations. Limnol. Oceanogr. 46, 1378–1391. 10.4319/lo.2001.46.6.1378

[B20] Cardol P. (2011). Mitochondrial NADH:ubiquinone oxidoreductase (complex I) in eukaryotes: a highly conserved subunit composition highlighted by mining of protein databases. Biochim. Biophys. Acta 1807, 1390–1397. 10.1016/j.bbabio.2011.06.015 21749854

[B21] Carreto J. I. Catoggio J. A. (1976). Variations in pigment contents of the diatom *Phaeodactylum tricornutum* during growth. Mar. Biol. 36, 105–112. 10.1007/BF00388433

[B22] Castruita M. Casero D. Karpowicz S. J. Kropat J. Vieler A. Hsieh S. I. (2011). Systems biology approach in *Chlamydomonas* reveals connections between copper nutrition and multiple metabolic steps. Plant Cell 23, 1273–1292. 10.1105/tpc.111.084400 21498682 PMC3101551

[B23] Cavalier-Smith T. Chao E. E. Y. (2006). Phylogeny and megasystematics of phagotrophic heterokonts (kingdom Chromista). J. Mol. Evol. 62, 388–420. 10.1007/s00239-004-0353-8 16557340

[B24] Cavalier-Smith T. (2004). “Chromalveolate diversity and cell megaevolution: interplay of membranes, genomes and cytoskeleton,” in Organelles, genomes and eukaryote phylogeny: an evolutionary synthesis in the age of genomics. Eds. Hirt R. P. Horner D. S. (London, UK: CRC Press LLC), 75–108.

[B25] Chauton M. S. Winge P. Brembu T. Vadstein O. Bones A. M. (2013). Gene regulation of carbon fixation, storage, and utilization in the diatom *Phaeodactylum tricornutum* acclimated to light/dark cycles. Plant Physiol. 161, 1034–1048. 10.1104/pp.112.206177 23209127 PMC3561001

[B26] Chen Z. Yang M. K. Li C. Y. Wang Y. Zhang J. Wang D. B. (2014). Phosphoproteomic analysis provides novel insights into stress responses in *Phaeodactylum tricornutum*, a model diatom. J. Proteome. Res. 13, 2511–2523. 10.1021/pr401290u 24712722

[B27] Chen X.-H. Li Y.-Y. Zhang H. Liu J.-L. Xie Z.-X. Lin L. (2018). Quantitative proteomics reveals common and specific responses of a marine diatom *Thalassiosira pseudonana* to different macronutrient deficiencies. Front. Microbiol. 9. 10.3389/fmicb.2018.02761 PMC6246746 30487787

[B28] Clement R. Jensen E. Prioretti L. Maberly S. C. Gontero B. (2017a). Diversity of CO_2_-concentrating mechanisms and responses to CO_2_ concentration in marine and freshwater diatoms. J. Exp. Bot. 68, 3925–3935. 10.1093/jxb/erx035 28369472

[B29] Clement R. Lignon S. Mansuelle P. Jensen E. Pophillat M. Lebrun R. (2017b). Responses of the marine diatom *Thalassiosira pseudonana* to changes in CO*_2_* concentration: a proteomic approach. Sci. Rep. 7. 10.1038/srep42333 PMC5299434 28181560

[B30] Colman B. Rotatore C. (1988). Uptake and accumulation of inorganic carbon by a fresh-water diatom. J. Exp. Bot. 39, 1025–1032. 10.1093/jxb/39.8.1025

[B31] Colman B. Rotatore C. (1995). Photosynthetic inorganic carbon uptake and accumulation in two marine diatoms. Plant Cell Environ. 18, 919–924. 10.1111/j.1365-3040.1995.tb00601.x

[B32] Daboussi F. Leduc S. Marechal A. Dubois G. Guyot V. Perez-Michaut C. (2014). Genome engineering empowers the diatom *Phaeodactylum tricornutum* for biotechnology. Nat. Commun. 5, 3831. 10.1038/ncomms4831 24871200

[B33] Del Prete S. Nocentini A. Supuran C. T. Capasso C. (2020). Bacterial iota-carbonic anhydrase: a new active class of carbonic anhydrase identified in the genome of the Gram-negative bacterium *Burkholderia territorii*. J. Enzyme Inhib. Med. Chem. 35, 1060–1068. 10.1080/14756366.2020.1755852 32314608 PMC7191908

[B34] Deschamps P. Moreira D. (2012). Reevaluating the green contribution to diatom genomes. Genome Biol. Evol. 4, 795–800. 10.1093/gbe/evs053 PMC5635612 22684208

[B35] Dickson A. G. (2010). “The carbon dioxide system in seawater: equilibrium chemistry and measurements,” in Guide to best practices for ocean acidification research and data reporting. Eds. Riebesell U. Fabry V. J. Hansson L. Gattuso J. P. (Luxembourg: Publications Office of the European Union), 17–40.

[B36] Dimario R. J. Machingura M. C. Waldrop G. L. Moroney J. V. (2018). The many types of carbonic anhydrases in photosynthetic organisms. Plant Sci. 268, 11–17. 10.1016/j.plantsci.2017.12.002 29362079

[B37] Dorrell R. G. Gile G. Mccallum G. Meheust R. Bapteste E. P. Klinger C. M. (2017). Chimeric origins of ochrophytes and haptophytes revealed through an ancient plastid proteome. Elife 6, e23717. 10.7554/eLife.23717 28498102 PMC5462543

[B38] Dupont C. L. Goepfert T. J. Lo P. Wei L. P. Ahnerz B. A. (2004). Diurnal cycling of glutathione in marine phytoplankton: field and culture studies. Limnol. Oceanogr. 49, 991–996. 10.4319/lo.2004.49.4.0991

[B39] Dutkiewicz S. Morris J. J. Follows M. J. Scott J. Levitan O. Dyhrman S. T. (2015). Impact of ocean acidification on the structure of future phytoplankton communities. Nat. Clim. Change 5, 1002–1006. 10.1038/nclimate2722

[B40] Ewe D. Tachibana M. Kikutani S. Gruber A. Bartulos C. R. Konert G. (2018). The intracellular distribution of inorganic carbon fixing enzymes does not support the presence of a C_4_ pathway in the diatom *Phaeodactylum tricornutum*. Photosynth. Res. 137, 263–280. 10.1007/s11120-018-0500-5 29572588

[B41] Fabris M. Matthijs M. Rombauts S. Vyverman W. Goossens A. Baart G. J. E. (2012). The metabolic blueprint of *Phaeodactylum tricornutum* reveals a eukaryotic Entner-Doudoroff glycolytic pathway. Plant J. 70, 1004–1014. 10.1111/j.1365-313X.2012.04941.x 22332784

[B42] Falciatore A. Jaubert M. Bouly J.-P. Bailleul B. Mock T. (2020). Diatom molecular research comes of age: model species for studying phytoplankton biology and diversity. Plant Cell 32, 547–572. 10.1105/tpc.19.00158 31852772 PMC7054031

[B43] Falkowski P. G. Owens T. G. (1980). Light-shade adaptation - 2 strategies in marine-phytoplankton. Plant Physiol. 66, 592–595. 10.1104/pp.66.4.592 16661484 PMC440685

[B44] Falkowski P. G. Barber R. T. Smetacek V. (1998). Biogeochemical controls and feedbacks on ocean primary production. Science 281, 200–206. 10.1126/science.281.5374.200 9660741

[B45] Fey V. Wagner R. Brautigam K. Pfannschmidt T. (2005). Photosynthetic redox control of nuclear gene expression. J. Exp. Bot. 56, 1491–1498. 10.1093/jxb/eri180 15863445

[B46] Fournier M. L. Paulson A. Pavelka N. Mosley A. L. Gaudenz K. Bradford W. D. (2010). Delayed correlation of mRNA and protein expression in rapamycin-treated cells and a role for Ggc1 in cellular sensitivity to rapamycin. Mol. Cell. Proteomics 9, 271–284. 10.1074/mcp.M900415-MCP200 19955083 PMC2830839

[B47] Gao K. Campbell D. A. (2014). Photophysiological responses of marine diatoms to elevated CO_2_ and decreased pH: a review. Funct. Plant Biol. 41, 449–459. 10.1071/FP13247 32481004

[B48] Gardemann A. Stitt M. Heldt H. W. (1983). Control of CO_2_ fixation. Regulation of spinach ribulose-5-phosphate kinase by stromal metabolite levels. BBA-Bioenergetics 722, 51–60. 10.1016/0005-2728(83)90156-1

[B49] Gattuso J. P. Magnan A. Billé R. Cheung W. W. L. Howes E. L. Joos F. (2015). Contrasting futures for ocean and society from different anthropogenic CO_2_ emissions scenarios. Science 349, aac4722. 10.1126/science.aac4722780 26138982

[B50] Gilstad M. Johnsen G. Sakshaug E. (1993). Photosynthetic parameters, pigment composition and respiration rates of the marine diatom *Skeletonema-costatum* grown in continuous light and a 12/12 h light-dark cycle. J. Plankton Res. 15, 939–951. 10.1093/plankt/15.8.939

[B51] Giordano M. Chen Y. B. Koblizek M. Falkowski P. G. (2005). Regulation of nitrate reductase in *Chlamydomonas reinhardtii* by the redox state of the plastoquinone pool. Eur. J. Phycol. 40, 345–352. 10.1080/09670260500334263

[B52] Gontero B. Avilan L. Lebreton S. (2007). “Control of carbon fixation in chloroplasts,” in Annual plant reviews. Eds. Plaxton W. C. Mcmanus M. T. (Oxford: Blackwell Publishing), 187–218. 10.1002/9780470988640.ch7

[B53] Grabsztunowicz M. Koskela M. M. Mulo P. (2017). Post-translational modifications in regulation of chloroplast function: recent advances. Front. Plant Sci. 8, 1–12. 10.3389/fpls.2017.00240 28280500 PMC5322211

[B54] Green B. R. (2011). After the primary endosymbiosis: an update on the chromalveolate hypothesis and the origins of algae with Chl *c*. Photosynth. Res. 107, 103–115. 10.1007/s11120-010-9584-2 20676772

[B55] Gruber A. Weber T. Bártulos C. R. Vugrinec S. Kroth P. G. (2009). Intracellular distribution of the reductive and oxidative pentose phosphate pathways in two diatoms. J. Basic Microb. 49, 58–72. 10.1002/jobm.200800339 19206144

[B56] Haimovich-Dayan M. Garfinkel N. Ewe D. Marcus Y. Gruber A. Wagner H. (2013). The role of C_4_ metabolism in the marine diatom *Phaeodactylum tricornutum*. New Phytol. 197, 177–185. 10.1111/j.1469-8137.2012.04375.x 23078356

[B57] Harada H. Matsuda Y. (2005). Identification and characterization of a new carbonic anhydrase in the marine diatom *Phaeodactylum tricornutum*. Can. J. Bot. 83, 909–916. 10.1139/b05-078

[B58] Harada H. Nakatsuma D. Ishida M. Matsuda Y. (2005). Regulation of the expression of intracellular beta-carbonic anhydrase in response to CO_2_ and light in the marine diatom *Phaeodactylum tricornutum*. Plant Physiol. 139, 1041–1050. 10.1104/pp.105.065185 16169965 PMC1256016

[B59] Harada H. Nakajima K. Sakaue K. Matsuda Y. (2006). CO_2_ sensing at ocean surface mediated by cAMP in a marine diatom. Plant Physiol. 142, 1318–1328. 10.1104/pp.106.086561 17012409 PMC1630750

[B60] Harrison P. J. Thompson P. A. Calderwood G. S. (1990). Effects of nutrient and light limitation on the biochemical composition of phytoplankton. J. Appl. Phycol. 2, 45–56. 10.1007/BF02179768

[B61] Hauser M. Eichelmann H. Oja V. Heber U. Laisk A. (1995). Stimulation by light of rapid pH regulation in the chloroplast stroma *in vivo* as indicated by CO_2_ solubilization in leaves. Plant Physiol. 108, 1059. 10.1104/pp.108.3.1059 12228527 PMC157457

[B62] Hennon G. M. M. Ashworth J. Groussman R. D. Berthiaume C. Morales R. L. Baliga N. S. (2015). Diatom acclimation to elevated CO_2_ via cAMP signalling and coordinated gene expression. Nat. Clim. Change 5, 761–765. 10.1038/nclimate2683

[B63] Herve V. Derr J. Douady S. Quinet M. Moisan L. Lopez P. J. (2012). Multiparametric analyses reveal the pH-dependence of silicon biomineralization in diatoms. PloS One 7, e46722. 10.1371/journal.pone.0046722 23144697 PMC3483172

[B64] Hesse S. J. A. Ruijter G. J. G. Dijkema C. Visser J. (2000). Measurement of intracellular (compartmental) pH by ^31^P NMR in. Aspergillus Niger J. Biotechnol. 77 , 5–15. 10.1016/S0168-1656(99)00203-5 10674210

[B65] Heydarizadeh P. Boureba W. Zahedi M. Huang B. Moreau B. Lukomska E. (2017). Response of CO_2_-starved diatom *Phaeodactylum tricornutum* to light intensity transition. Philos. Trans. R. Soc. B Biol. Sci. 372, 20160396. 10.1098/rstb.2016.0396 PMC5516105 28717022

[B66] Heydarizadeh P. Veidl B. Huang B. Lukomska E. Wielgosz-Collin G. Couzinet-Mossion A. (2019). Carbon orientation in the diatom *Phaeodactylum tricornutum*: the effects of carbon limitation and photon flux density. Front. Plant Sci. 10. 10.3389/fpls.2019.00471 PMC6477932 31057578

[B67] Hildebrand M. Manandhar-Shrestha K. Abbriano R. (2017). Effects of chrysolaminarin synthase knockdown in the diatom *Thalassiosira pseudonana*: Implications of reduced carbohydrate storage relative to green algae. Algal Res. 23, 66–77. 10.1016/j.algal.2017.01.010

[B68] Hildebrand M. Lerch S. J. L. Shrestha R. P. (2018). Understanding diatom cell wall silicification—moving forward. Front. Mar. Sci. 5. 10.3389/fmars.2018.00125

[B69] Hitchcock G. L. (1980). Diel variation in chlorophyll *a*, carbohydrate and protein content of the marine diatom *Skeletonema costatum*. Mar. Biol. 57, 271–278. 10.1007/BF00387570

[B70] Hopes A. Nekrasov V. Kamoun S. Mock T. (2016). Editing of the urease gene by CRISPR-Cas in the diatom Thalassiosira pseudonana. Plant Methods 12, 49. 10.1186/s13007-016-0148-0 27904648 PMC5121945

[B71] Hopkinson B. M. Dupont C. L. Allen A. E. Morel F. M. M. (2011). Efficiency of the CO_2_-concentrating mechanism of diatoms. Proc. Natl. Acad. Sci. U.S.A. 108, 3830–3837. 10.1073/pnas.1018062108 21321195 PMC3054024

[B72] Hopkinson B. M. Dupont C. L. Matsuda Y. (2016). The physiology and genetics of CO_2_ concentrating mechanisms in model diatoms. Curr. Opin. Plant Biol. 31, 51–57. 10.1016/j.pbi.2016.03.013 27055267

[B73] Huang W. Daboussi F. (2017). Genetic and metabolic engineering in diatoms. Philos. Trans. R. Soc. B Biol. Sci. 372, 20160411. 10.1098/rstb.2016.0411 PMC5516120 28717021

[B74] Huang W. Haferkamp I. Lepetit B. Molchanova M. Hou S. Jeblick W. (2018). Reduced vacuolar β-1,3-glucan synthesis affects carbohydrate metabolism as well as plastid homeostasis and structure in *Phaeodactylum tricornutum*. Proc. Natl. Acad. Sci. U.S.A. 115, 4791–4796. 10.1073/pnas.1719274115 29669920 PMC5939080

[B75] Huysman M. J. J. Fortunato A. E. Matthijs M. Costa B. S. Vanderhaeghen R. Van Den Daele H. (2013). AUREOCHROME1a-mediated induction of the diatom-specific cyclin dsCYC2 controls the onset of cell division in diatoms (*Phaeodactylum tricornutum*). Plant Cell 25, 215–228. 10.1105/tpc.112.106377 23292736 PMC3584536

[B76] IPCC (2014). “Climate Change. Synthesis Report,” in Contribution of Working Groups I, II and III to the Fifth Assessment Report of the Intergovernmental Panel on Climate Change, vol. 151 Eds. Core Writing Team Pachauri R. K. Meyer L. A. (Geneva, Switzerland: IPCC).

[B77] Jensen E. Clement R. Maberly S. C. Gontero B. (2017). Regulation of the Calvin-Benson-Bassham cycle in the enigmatic diatoms: biochemical and evolutionary variations on an original theme. Philos. Trans. R. Soc. B Biol. Sci. 372, 20160401. 10.1098/rstb.2016.0401 PMC5516110 28717027

[B78] Jensen E. L. Clement R. Kosta A. Maberly S. C. Gontero B. (2019a). A new widespread subclass of carbonic anhydrase in marine phytoplankton. ISME J. 13, 2094–2106. 10.1038/s41396-019-0426-8 31024153 PMC6776030

[B79] Jensen E. L. Yanguez K. Carriere F. Gontero B. (2019b). Storage compound accumulation in diatoms as response to elevated CO_2_ concentration. Biology 9, 5. 10.3390/biology9010005 PMC7169399 31878202

[B80] Jensen E. L. Maberly S. C. Gontero B. (2020). Insights on the functions and ecophysiological relevance of the diverse carbonic anhydrases in microalgae. Int. J. Mol. Sci. 21, 2922. 10.3390/ijms21082922 PMC7215798 32331234

[B81] Keeling P. J. (2009). Chromalveolates and the evolution of plastids by secondary endosymbiosis. J. Eukaryot. Microbiol. 56, 1–8. 10.1111/j.1550-7408.2008.00371.x 19335769

[B82] Kikutani S. Tanaka R. Yamazaki Y. Hara S. Hisabori T. Kroth P. G. (2012). Redox regulation of carbonic anhydrases via thioredoxin in chloroplast of the marine diatom *Phaeodactylum tricornutum*. J. Biol. Chem. 287, 20689–20700. 10.1074/jbc.M111.322743 22535967 PMC3370251

[B83] Kim J.-M. Lee K. Shin K. Kang J.-H. Lee H.-W. Kim M. (2006). The effect of seawater CO_2_ concentration on growth of a natural phytoplankton assemblage in a controlled mesocosm experiment. Limnol. Oceanogr. 51, 1629–1636. 10.4319/lo.2006.51.4.1629

[B84] Knuesting J. Scheibe R. (2018). Small molecules govern thiol redox switches. Trends Plant Sci. 23, 769–782. 10.1016/j.tplants.2018.06.007 30149854

[B85] Kojadinovic-Sirinelli M. Villain A. Puppo C. Sing S. F. Prioretti L. Hubert P. (2018). Exploring the microbiome of the “star” freshwater diatom *Asterionella formosa* in a laboratory context. Environ. Microbiol. 20, 3601–3615. 10.1111/1462-2920.14337 30063098

[B86] Kroth P. G. Chiovitti A. Gruber A. Martin-Jezequel V. Mock T. Parker M. S. (2008). A model for carbohydrate metabolism in the diatom *Phaeodactylum tricornutum* deduced from comparative whole genome analysis. PLoS One 3, e1426. 10.1371/journal.pone.0001426 18183306 PMC2173943

[B87] Kroth P. G. Bones A. M. Daboussi F. Ferrante M. I. Jaubert M. Kolot M. (2018). Genome editing in diatoms: achievements and goals. Plant Cell. Rep. 37, 1401–1408. 10.1007/s00299-018-2334-1 30167805

[B88] Kuczynska P. Jemiola-Rzeminska M. Strzalka K. (2015). Photosynthetic pigments in diatoms. Mar. Drugs 13, 5847–5881. 10.3390/md13095847 26389924 PMC4584358

[B89] Kustka A. B. Milligan A. J. Zheng H. New A. M. Gates C. Bidle K. D. (2014). Low CO_2_ results in a rearrangement of carbon metabolism to support C_4_ photosynthetic carbon assimilation in *Thalassiosira pseudonana*. New Phytol. 204, 507–520. 10.1111/nph.12926 25046577

[B90] Lauritano C. Ferrante M. I. Rogato A. (2019). Marine natural products from microalgae: an -omics overview. Mar. Drugs 17, 269. 10.3390/md17050269 PMC6562964 31067655

[B91] Lavaud J. Materna A. C. Sturm S. Vugrinec S. Kroth P. G. (2012). Silencing of the violaxanthin de-epoxidase gene in the diatom *Phaeodactylum tricornutum* reduces diatoxanthin synthesis and non-photochemical quenching. PloS One 7, e36806. 10.1371/journal.pone.0036806 22629333 PMC3356336

[B92] Lin Q. Liang J. R. Huang Q. Q. Luo C. S. Anderson D. M. Bowler C. (2017). Differential cellular responses associated with oxidative stress and cell fate decision under nitrate and phosphate limitations in *Thalassiosira pseudonana*: Comparative proteomics. PloS One 12, e0184849. 10.1371/journal.pone.0184849 28910417 PMC5599023

[B93] Lohr M. Wilhelm C. (1999). Algae displaying the diadinoxanthin cycle also possess the violaxanthin cycle. Proc. Natl. Acad. Sci. U.S.A. 96, 8784–8789. 10.1073/pnas.96.15.8784 10411953 PMC17594

[B94] Loiselle F. B. Casey J. R. (2010). “Measurement of intracellular pH,” in Membrane transporters in drug discovery and development: methods and protocols. Ed. Yan Q. (Totowa, NJ: Humana Press), 311–331.

[B95] Maberly S. C. Gontero B. (2017). Ecological imperatives for aquatic CO_2_-concentrating mechanisms. J. Exp. Bot. 68, 3797–3814. 10.1093/jxb/erx201 28645178

[B96] Maberly S. C. Courcelle C. Groben R. Gontero B. (2010). Phylogenetically-based variation in the regulation of the Calvin cycle enzymes, phosphoribulokinase and glyceraldehyde-3-phosphate dehydrogenase, in algae. J. Exp. Bot. 61, 735–745. 10.1093/jxb/erp337 19926682

[B97] Maier T. Güell M. Serrano L. (2009). Correlation of mRNA and protein in complex biological samples. FEBS Lett. 583, 3966–3973. 10.1016/j.febslet.2009.10.036 19850042

[B98] Mann D. G. Vanormelingen P. (2013). An inordinate fondness? The number, distributions, and origins of diatom species. J. Eukaryot. Microbiol. 60, 414–420. 10.1111/jeu.12047 23710621

[B99] Marri L. Thieulin-Pardo G. Lebrun R. Puppo R. Zaffagnini M. Trost P. (2014). CP12-mediated protection of Calvin-Benson cycle enzymes from oxidative stress. Biochimie 97, 228–237. 10.1016/j.biochi.2013.10.018 24211189

[B100] Matsuda Y. Kroth P. G. (2014). ““Carbon fixation in diatoms,”,” in The structural basis of biological energy generation. Advances in photosynthesis and respiration (including bioenergy and related processes). Ed. Hohmann-Marriott M. F. (Dordrecht: Springer Netherlands), 335–362.

[B101] Matsuda Y. Nakajima K. Tachibana M. (2011). Recent progresses on the genetic basis of the regulation of CO_2_ acquisition systems in response to CO_2_ concentration. Photosynth. Res. 109, 191–203. 10.1007/s11120-011-9623-7 21287273

[B102] Matsuda Y. Hopkinson B. M. Nakajima K. Dupont C. L. Tsuji Y. (2017). Mechanisms of carbon dioxide acquisition and CO_2_ sensing in marine diatoms: a gateway to carbon metabolism. Philos. Trans. R. Soc. B Biol. Sci. 372, 20160403. 10.1098/rstb.2016.0403 PMC5516112 28717013

[B103] Medlin L. K. (2016). Evolution of the diatoms: major steps in their evolution and a review of the supporting molecular and morphological evidence. Phycologia 55, 79–103. 10.2216/15-105.1

[B104] Mekhalfi M. Avilan L. Lebrun R. Botebol H. Gontero B. (2012). Consequences of the presence of 24-epibrassinolide, on cultures of a diatom, *Asterionella formosa*. Biochimie 94, 1213–1220. 10.1016/j.biochi.2012.02.011 22586703

[B105] Mekhalfi M. Puppo C. Avilan L. Lebrun R. Mansuelle P. Maberly S. C. (2014). Glyceraldehyde-3-phosphate dehydrogenase is regulated by ferredoxin-NADP reductase in the diatom *Asterionella formosa*. New Phytol. 203, 414–423. 10.1111/nph.12820 24799178

[B106] Michels A. K. Wedel N. Kroth P. G. (2005). Diatom plastids possess a phosphoribulokinase with an altered regulation and no oxidative pentose phosphate pathway. Plant Physiol. 137, 911–920. 10.1104/pp.104.055285 15734914 PMC1065392

[B107] Mock T. Otillar R. P. Strauss J. Mcmullan M. Paajanen P. Schmutz J. (2017). Evolutionary genomics of the cold-adapted diatom Fragilariopsis cylindrus. Nature 541, 536–540. 10.1038/nature20803 28092920

[B108] Montrichard F. Alkhalfioui F. Yano H. Vensel W. H. Hurkman W. J. Buchanan B. B. (2009). Thioredoxin targets in plants: the first 30 years. J. Proteomics 72, 452–474. 10.1016/j.jprot.2008.12.002 19135183

[B109] Morel F. M. M. Lam P. J. Saito M. A. (2020). Trace metal substitution in marine phytoplankton. Annu. Rev. Earth Planet. Sci. 48, 491–517. 10.1146/annurev-earth-053018-060108

[B110] Moustafa A. Beszteri B. Maier U. G. Bowler C. Valentin K. Bhattacharya D. (2009). Genomic footprints of a cryptic plastid endosymbiosis in diatoms. Science 324, 1724–1726. 10.1126/science.1172983 19556510

[B111] Mueller-Cajar O. Stotz M. Wendler P. Hartl F. U. Bracher A. Hayer-Hartl M. (2011). Structure and function of the AAA^+^ protein CbbX, a red-type Rubisco activase. Nature 479, 194–199. 10.1038/nature10568 22048315

[B112] Muhseen Z. T. Xiong Q. Chen Z. Ge F. (2015). Proteomics studies on stress responses in diatoms. Proteomics 15, 3943–3953. 10.1002/pmic.201500165 26364674

[B113] Nakajima K. Tanaka A. Matsuda Y. (2013). SLC4 family transporters in a marine diatom directly pump bicarbonate from seawater. Proc. Natl. Acad. Sci. U.S.A. 110, 1767–1772. 10.1073/pnas.1216234110 23297242 PMC3562803

[B114] Nikolaev V. O. Lohse M. J. (2006). Monitoring of cAMP synthesis and degradation in living cells. Physiology 21, 86–92. 10.1152/physiol.00057.2005 16565474

[B115] Nonoyama T. Kazamia E. Nawaly H. Gao X. Tsuji Y. Matsuda Y. (2019). Metabolic innovations underpinning the origin and diversification of the diatom chloroplast. Biomolecules 9, 322. 10.3390/biom9080322 PMC6723447 31366180

[B116] Norici A. Bazzoni A. M. Pugnetti A. Raven J. A. Giordano M. (2011). Impact of irradiance on the C allocation in the coastal marine diatom *Skeletonema marinoi* Sarno and Zingone. Plant Cell Environ. 34, 1666–1677. 10.1111/j.1365-3040.2011.02362.x 21707652

[B117] Nymark M. Sharma A. K. Sparstad T. Bones A. M. Winge P. (2016). A CRISPR/Cas9 system adapted for gene editing in marine algae. Sci. Rep. 6, 24951. 10.1038/srep24951 27108533 PMC4842962

[B118] Ogura A. Akizuki Y. Imoda H. Mineta K. Gojobori T. Nagai S. (2018). Comparative genome and transcriptome analysis of diatom, *Skeletonema costatum*, reveals evolution of genes for harmful algal bloom. BMC Genomics 19, 765. 10.1186/s12864-018-5144-5 30348078 PMC6198448

[B119] Ohno N. Inoue T. Yamashiki R. Nakajima K. Kitahara Y. Ishibashi M. (2012). CO_2_-cAMP-responsive cis-elements targeted by a transcription factor with CREB/ATF-Like basic zipper domain in the marine diatom *Phaeodactylum tricornutum*. Plant Physiol. 158, 499–513. 10.1104/pp.111.190249 22095044 PMC3252111

[B120] Oudot-Le Secq M. P. Grimwood J. Shapiro H. Armbrust E. V. Bowler C. Green B. R. (2007). Chloroplast genomes of the diatoms *Phaeodactylum tricornutum* and *Thalassiosira pseudonana*: comparison with other plastid genomes of the red lineage. Mol. Genet. Genomics 277, 427–439. 10.1007/s00438-006-0199-4 17252281

[B121] Perez-Perez M. E. Mauries A. Maes A. Tourasse N. J. Hamon M. Lemaire S. D. (2017). The deep thioredoxome in *Chlamydomonas reinhardtii*: new insights into redox regulation. Mol. Plant 10, 1107–1125. 10.1016/j.molp.2017.07.009 28739495

[B122] Ponnala L. Wang Y. Sun Q. Van Wijk K. J. (2014). Correlation of mRNA and protein abundance in the developing maize leaf. Plant J. 78, 424–440. 10.1111/tpj.12482 24547885

[B123] Portis A. R. Heldt H. W. (1976). Light-dependent changes of Mg^2+^ concentration in stroma in relation to Mg^2+^ dependency of CO_2_ fixation in intact chloroplasts. BBA-Bioenergetics 449, 434–446. 10.1016/0005-2728(76)90154-7 11816

[B124] Prabakaran S. Lippens G. Steen H. Gunawardena J. (2012). Post-translational modification: nature’s escape from genetic imprisonment and the basis for dynamic information encoding. Wires Syst. Biol. Med. 4, 565–583. 10.1002/wsbm.1185 PMC3473174 22899623

[B125] Prosser G. A. Larrouy-Maumus G. De Carvalho L. P. (2014). Metabolomic strategies for the identification of new enzyme functions and metabolic pathways. EMBO Rep. 15, 657–669. 10.15252/embr.201338283 24829223 PMC4197876

[B126] Pupillo P. Giulianipiccari G. (1975). The reversible depolymerization of spinach chloroplast glyceraldehyde-phosphate dehydrogenase-interaction with nucleotides and dithiothreitol. Eur. J. Biochem. 51, 475–482. 10.1111/j.1432-1033.1975.tb03947.x 238837

[B127] Raven J. Brown K. Mackay M. Beardall J. Giordano M. Granum E. (2005). “Iron, nitrogen, phosphorus and zinc cycling and consequences for primary productivity in the oceans,” in Micro-organisms and earth systems-advances in geomicrobiology. Eds. Gadd G. Semple K. Lappin-Scott H. (Cambridge, UK: Cambridge University Press), 247–272.

[B128] Raven J. A. (1994). Carbon fixation and carbon availability in marine phytoplankton. Photosynth. Res. 39, 259–273. 10.1007/BF00014587 24311125

[B129] Reinfelder J. R. Kraepiel A. M. L. Morel F. M. M. (2000). Unicellular C_4_ photosynthesis in a marine diatom. Nature 407, 996–999. 10.1038/35039612 11069177

[B130] Reinfelder J. R. (2011). “Carbon concentrating mechanisms in eukaryotic marine phytoplankton,” in Annu. Rev. Mar. Sci. Eds. Carlson C. A. Giovannoni S. J. (Palo Alto, USA: Annual Reviews Publisher), 291–315. 10.1146/annurev-marine-120709-142720 21329207

[B131] Riebesell U. Wolfgladrow D. A. Smetacek V. (1993). Carbon dioxide limitation of marine phytoplankton growth rates. Nature 361, 249–251. 10.1038/361249a0

[B132] Rosenwasser S. Graff Van Creveld S. Schatz D. Malitsky S. Tzfadia O. Aharoni A. (2014). Mapping the diatom redox-sensitive proteome provides insight into response to nitrogen stress in the marine environment. Proc. Natl. Acad. Sci. U.S.A. 111, 2740–2745. 10.1073/pnas.1319773111 24550302 PMC3932894

[B133] Sage R. F. (2004). The evolution of C_4_ photosynthesis. New Phytol. 161, 341–370. 10.1111/j.1469-8137.2004.00974.x 33873498

[B134] Samukawa M. Shen C. Hopkinson B. M. Matsuda Y. (2014). Localization of putative carbonic anhydrases in the marine diatom, Thalassiosira pseudonana. Photosynth. Res. 121, 235–249. 10.1007/s11120-014-9967-x 24414291

[B135] Santin Y. G. Doan T. Lebrun R. Espinosa L. Journet L. Cascales E. (2018). In vivo TssA proximity labelling during type VI secretion biogenesis reveals TagA as a protein that stops and holds the sheath. Nat. Microbiol. 3, 1304–1313. 10.1038/s41564-018-0234-3 30275513

[B136] Satoh D. Hiraoka Y. Colman B. Matsuda Y. (2001). Physiological and molecular biological characterization of intracellular carbonic anhydrase from the marine diatom *Phaeodactylum tricornutum*. Plant Physiol. 126, 1459–1470. 10.1104/pp.126.4.1459 11500545 PMC117146

[B137] Schoefs B. Hu H. Kroth P. G. (2017). The peculiar carbon metabolism in diatoms. Philos. Trans. R. Soc. B Biol. Sci. 372, 20160405. 10.1098/rstb.2016.0405 PMC5516114 28717015

[B138] Schurmann P. Jacquot J. P. (2000). Plant thioredoxin systems revisited. Annu. Rev. Plant Physiol. Plant Mol. Biol. 51, 371–400. 10.1146/annurev.arplant.51.1.371 15012197

[B139] Stein K. C. Frydman J. (2019). The stop-and-go traffic regulating protein biogenesis: how translation kinetics controls proteostasis. J. Biol. Chem. 294, 2076–2084. 10.1074/jbc.REV118.002814 30504455 PMC6369277

[B140] Stiller J. W. Schreiber J. Yue J. Guo H. Ding Q. Huang J. (2014). The evolution of photosynthesis in chromist algae through serial endosymbioses. Nat. Commun. 5, 5764. 10.1038/ncomms6764 25493338 PMC4284659

[B141] Subramanian S. Barry A. N. Pieris S. Sayre R. T. (2013). Comparative energetics and kinetics of autotrophic lipid and starch metabolism in chlorophytic microalgae: implications for biomass and biofuel production. Biotechnol. Biofuels 6, 150. 10.1186/1754-6834-6-150 24139286 PMC4015678

[B142] Sun N. Ma L. G. Pan D. Y. Zhao H. Y. Deng X. W. (2003). Evaluation of light regulatory potential of Calvin cycle steps based on large-scale gene expression profiling data. Plant Mol. Biol. 53, 467–478. 10.1023/B:PLAN.0000019071.12878.9e 15010613

[B143] Tachibana M. Allen A. E. Kikutani S. Endo Y. Bowler C. Matsuda Y. (2011). Localization of putative carbonic anhydrases in two marine diatoms, *Phaeodactylum tricornutum* and *Thalassiosira pseudonana*. Photosynth. Res. 109, 205–221. 10.1007/s11120-011-9634-4 21365259

[B144] Tanaka T. Maeda Y. Veluchamy A. Tanaka M. Abida H. Marechal E. (2015). Oil accumulation by the oleaginous diatom *Fistulifera solaris* as revealed by the genome and transcriptome. Plant Cell 27, 162–176. 10.1105/tpc.114.135194 25634988 PMC4330590

[B145] Tanaka A. Ohno N. Nakajima K. Matsuda Y. (2016). Light and CO_2_/cAMP signal cross talk on the promoter elements of chloroplastic beta-carbonic anhydrase genes in the marine diatom *Phaeodactylum tricornutum*. Plant Physiol. 170, 1105–1116. 10.1104/pp.15.01738 26662605 PMC4734587

[B146] Taraldsvik M. Myklestad S. M. (2000). The effect of pH on growth rate, biochemical composition and extracellular carbohydrate production of the marine diatom *Skeletonema costatum*. Eur. J. Phycol. 35, 189–194. 10.1080/09670260010001735781

[B147] Thieulin-Pardo G. Remy T. Lignon S. Lebrun R. Gontero B. (2015). Phosphoribulokinase from *Chlamydomonas reinhardtii*: a Benson-Calvin cycle enzyme enslaved to its cysteine residues. Mol. Biosyst. 11, 1134–1145. 10.1039/C5MB00035A 25688043

[B148] Torstensson A. Chierici M. Wulff A. (2012). The influence of increased temperature and carbon dioxide levels on the benthic/sea ice diatom *Navicula directa*. Polar Biol. 35, 205–214. 10.1007/s00300-011-1056-4

[B149] Tortell P. D. (2000). Evolutionary and ecological perspectives on carbon acquisition in phytoplankton. Limnol. Oceanogr. 45, 744–750. 10.4319/lo.2000.45.3.0744

[B150] Traller J. C. Cokus S. J. Lopez D. A. Gaidarenko O. Smith S. R. Mccrow J. P. (2016). Genome and methylome of the oleaginous diatom *Cyclotella cryptica* reveal genetic flexibility toward a high lipid phenotype. Biotechnol. Biofuels 9, 258. 10.1186/s13068-016-0670-3 27933100 PMC5124317

[B151] Tsuji Y. Nakajima K. Matsuda Y. (2017). Molecular aspects of the biophysical CO_2_-concentrating mechanism and its regulation in marine diatoms. J. Exp. Bot. 68, 3763–3772. 10.1093/jxb/erx173 28633304

[B152] Valegard K. Andralojc P. J. Haslam R. P. Pearce F. G. Eriksen G. K. Madgwick P. J. (2018). Structural and functional analyses of Rubisco from arctic diatom species reveal unusual posttranslational modifications. J. Biol. Chem. 293, 13033–13043. 10.1074/jbc.RA118.003518 29925588 PMC6109933

[B153] Valenzuela J. J. Garcia De Lomana A. L. Lee A. Armbrust E. V. Orellana M. V. Baliga N. S. (2018). Ocean acidification conditions increase resilience of marine diatoms. Nat. Commun. 9, 2328. 10.1038/s41467-018-04742-3 29899534 PMC5997998

[B154] Van Der Star W. R. Dijkema C. De Waard P. Picioreanu C. Strous M. Van Loosdrecht M. C. (2010). An intracellular pH gradient in the anammox bacterium *Kuenenia stuttgartiensis* as evaluated by ^31^P NMR. Appl. Microbiol. Biot. 86, 311–317. 10.1007/s00253-009-2309-9 PMC2822221 19862513

[B155] Villain A. Kojadinovic M. Puppo C. Prioretti L. Hubert P. Zhang Y. (2017). Complete mitochondrial genome sequence of the freshwater diatom *Asterionella formosa*. Mitochondrial DNA B 2, 97–98. 10.1080/23802359.2017.1285210 PMC7800273 33490441

[B156] Weber T. Gruber A. Kroth P. G. (2009). The presence and localization of thioredoxins in diatoms, unicellular algae of secondary endosymbiotic origin. Mol. Plant 2, 468–477. 10.1093/mp/ssp010 19825630

[B157] Wei L. El Hajjami M. Shen C. You W. Lu Y. Li J. (2019). Transcriptomic and proteomic responses to very low CO_2_ suggest multiple carbon concentrating mechanisms in *Nannochloropsis oceanica*. Biotechnol. Biofuels 12, 168. 10.1186/s13068-019-1506-8 31297156 PMC6599299

[B158] Werdan K. Heldt H. W. (1973). Measurement of pH value in stroma and thylakoid space of intact chloroplasts. Hoppe Seylers Z. Physiol. Chem. 354, 223–224.

[B159] Werdan K. Heldt H. W. Milovancev M. (1975). The role of pH in regulation of carbon fixation in chloroplast stroma. Studies on CO_2_ fixation in light and dark. BBA-Bioenergetics 396, 276–292. 10.1016/0005-2728(75)90041-9 239746

[B160] Wilhelm C. Buechel C. Fisahn J. Goss R. Jakob T. Laroche J. (2006). The regulation of carbon and nutrient assimilation in diatoms is significantly different from green algae. Protist 157, 91–124. 10.1016/j.protis.2006.02.003 16621693

[B161] Young J. N. Morel F. M. M. (2015). The CO_2_ switch in diatoms. Nat. Clim. Change 5, 722–723. 10.1038/nclimate2691

[B162] Young J. N. Heureux A. M. C. Sharwood R. E. Rickaby R. E. M. Morel F. M. M. Whitney S. M. (2016). Large variation in the Rubisco kinetics of diatoms reveals diversity among their carbon-concentrating mechanisms. J. Exp. Bot. 67, 3445–3456. 10.1093/jxb/erw163 27129950 PMC4892730

[B163] Zaffagnini M. Bedhomme M. Groni H. Marchand C. H. Puppo C. Gontero B. (2012). Glutathionylation in the photosynthetic model organism *Chlamydomonas reinhardtii*: a proteomic survey. Mol. Cell. Proteomics 11, M111.014142. 10.1074/mcp.M111.014142 PMC3277769 22122882

